# Optimization of technological parameters for durum wheat pasta production with carrot powder and ion-ozonated water

**DOI:** 10.1038/s41598-025-28027-0

**Published:** 2025-12-24

**Authors:** Galiya Iskakova, Bauyrzhan Iztayev, Auyelbek Iztayev, Talgat Kulazhanov, Berdan Rskeldiyev, Assel Izembayeva, Madina Yakiyayeva, Meruyet Baiysbayeva

**Affiliations:** Department of Technology of Bakery Products and Processing Industries, Almaty Technological University, 100 Tole bi str., Almaty, 050012 Kazakhstan

**Keywords:** Durum wheat, Wholemeal flour, Carrot powder, Ion-ozonated water, Pasta, Process modes, Quality, Multifactorial experimental design, Health care, Engineering

## Abstract

The quality of food products and a balanced diet play a key role in maintaining health and improving the quality of life. Due to the high popularity of pasta, a promising direction is the development of recipes that include whole grain flour, carrot powder and ion-ozonated water, which increase the nutritional value of the product and improve its chemical composition. The aim of this study was to optimise the technological modes of production of durum wheat pasta using carrot powder and ion-ozonated water to minimise the loss of dry matter (DM) during cooking and increase the protein content in the finished product. We used multifactorial experiment planning with the sequential regression analysis program PLAN. The variable factors considered were the concentration of ion-ozone in the water (C_io_), the carrot powder content (C_cp._), the temperature of the water (t_w_) used in kneading the dough and the drying temperature (t_d_). We found that a minimal DM loss into the cooking water (5.86%) was ensured using a C_cp._ of 3.0% and a t_d_ of 50 °C. Neither the C_io_ nor the t_w_ had any significant effect on this formula. To achieve the maximum protein content in the finished pasta (14.25%), the optimal parameters used were C_cp._ = 1.0% and t_d_ = 50 °C. Similarly, the C_io_ and t_w_ factors had no significant effect on the protein composition. These results demonstrate the possibility of controlling the quality indicators of pasta by regulating temperature parameters and the concentration of plant additives. To increase the nutritional value of pasta products without deteriorating the technological properties, it is recommended to use carrot powder at a concentration of 1.0–3.0% and dry it at 50 °C. The use of ion-ozonated water is justified from the point of view of safety and microbiological stability, but it did not have a significant impact on the quality indicators studied.

**Supplementary Information:**

The online version contains supplementary material available at 10.1038/s41598-025-28027-0.

## Introduction

Pasta remains one of the most popular staple foods globally due to its affordability, convenience, long shelf life, and balanced nutritional composition. Its traditional production relies on high-quality durum wheat semolina, which provides desirable sensory characteristics—firm texture, minimal cooking loss, and a golden-yellow colour^1–4^.

The semolina from Triticum durum is particularly valued for its high protein and gluten content, ensuring superior technological and cooking properties^5–7^. In recent years, there has been a growing demand for functional pasta products that not only satisfy taste preferences but also contribute to improved nutrition and health^8–10^. Consumers increasingly seek foods rich in fiber, antioxidants, vitamins, and minerals, which has led to intensified research in pasta enrichment using vegetable powders, whole grains, and natural bioactive compounds^11–13^.

Among such additives, carrot powder has received considerable attention as a natural source of β-carotene, vitamins A, C, and E, minerals such as potassium, calcium, and iron, and dietary fiber^14,15^. Numerous studies have shown that carrot powder can improve the antioxidant profile, colour, and nutritional value of pasta while maintaining or even enhancing its organoleptic properties. In optimal proportions (typically up to 10–15%), it does not negatively affect taste or texture, making it an ideal candidate for functional pasta formulations^16–19^.

However, the introduction of plant-based ingredients into pasta dough presents technological challenges^20–22^. These include changes in water absorption, gluten dilution, and modifications in dough rheology, often leading to reduced firmness or increased cooking loss. Therefore, to fully realize the benefits of functional additives, process optimization becomes essential, particularly concerning dough preparation and hydration^23–25^. One of the innovative aspects explored in this study is the application of ion-ozonated water during dough preparation^26,27^. While ozonated water has previously been studied for its antimicrobial and oxidative properties, ion-ozonated water provides additional functional advantages. It contains negatively charged oxygen ions (O₂⁻) in combination with ozone (O₃), which can enhance the biochemical interactions within the dough matrix, improve gluten development, and reduce microbial load without the need for chemical preservatives.

Compared to traditional processing, the use of ion-ozonated water offers several key benefits:

Enhanced microbiological safety: It reduces total microbial counts, which extends the shelf life of the product^28–30^.

Improved dough structure: Ion-ozonated water contributes to better hydration and cross-linking of gluten proteins, maintaining dough elasticity and integrity.

Retention of bioactive compounds: The mild, non-thermal nature of the treatment preserves heat-sensitive nutrients, such as carotenoids and vitamins, within the carrot powder.

Recent experimental data suggest that ion-ozonated water may counterbalance the adverse effects that fiber-rich plant additives can have on pasta quality. When properly applied, this method can stabilize dough properties, minimize losses during cooking, and support the overall functional and safety profile of the final product^31–33^.

Therefore, the integration of whole grain semolina from the ‘Satti’ variety, carrot powder as a natural enrichment, and ion-ozonated water represents a promising approach for developing innovative, functional pasta that meets modern consumer demands for healthier, high-quality food products.

To develop and optimize technological parameters for the production of functional pasta enriched with carrot powder using whole grain semolina from the Kazakh ‘Satti’ variety and ion-ozonated water, focusing on enhancing the nutritional value, improving sensory attributes, and ensuring microbiological safety and technological stability.

## Research results and discussion

Whole grain flour, obtained by grinding grains of the durum wheat ‘Satti’, and carrot powder were analysed. The organoleptic and physicochemical parameters of these raw materials were then determined, as provided in Table 1.

**Table 1:** Physical and chemical parameters of the Raw materials.

Indicator	Whole grain durum wheat ‘Satti’ flour	Carrot powder
Colour	Cream with a yellowish tinge	Orange
Smell	Characteristic of normal flour, with no smell of mould, mustiness or other foreign odours	Appropriate
Taste	Characteristic of normal flour, with no sour, bitter or other foreign flavours	Appropriate
Mineral impurities	No crunch when chewing the flour	Not detected
Humidity (%)	12.8	3.8
Raw gluten content (%)	30.6	–
Gluten quality from IDK-1, unit of the device	84	–
Fineness of grinding (%) Residue on the silk fabric sieve (%) Passage through the silk fabric sieve (%)	No. 23 sieve: 2 No. 35 sieve: 9	No. 35 sieve: 2.14 No. 43 sieve: 60.9
Ash content (DM, %)	1.61	3.57
Metal impurities content (mg/kg of flour)	–	Not detected
Pest infestation of grain stocks	Not detected	–

Based on the results of the analyses of the organoleptic and physicochemical parameters, both the whole wheat flour and carrot powder were found to meet the requirements of the regulatory and technical standards.

**Fig. 1:**
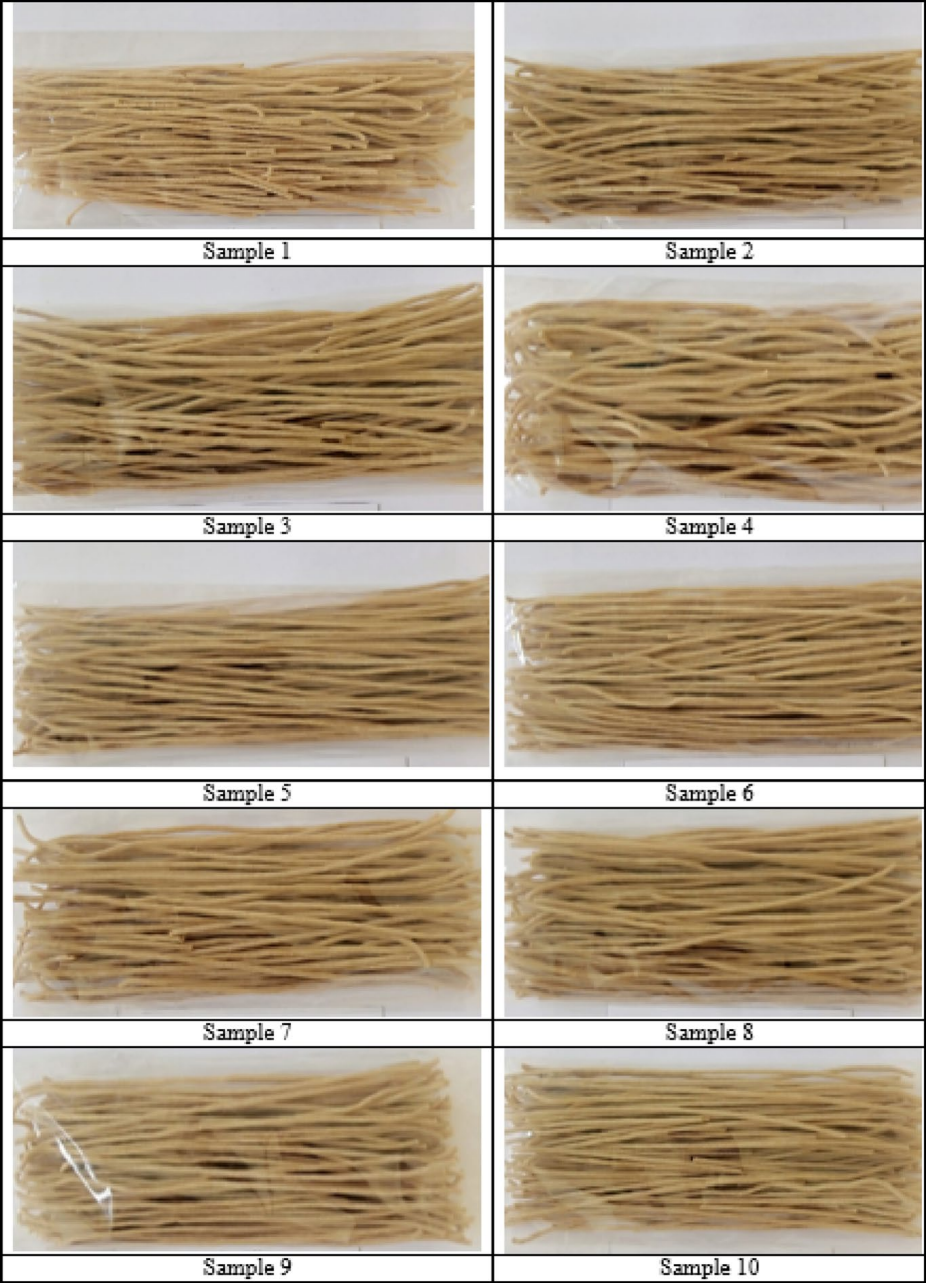

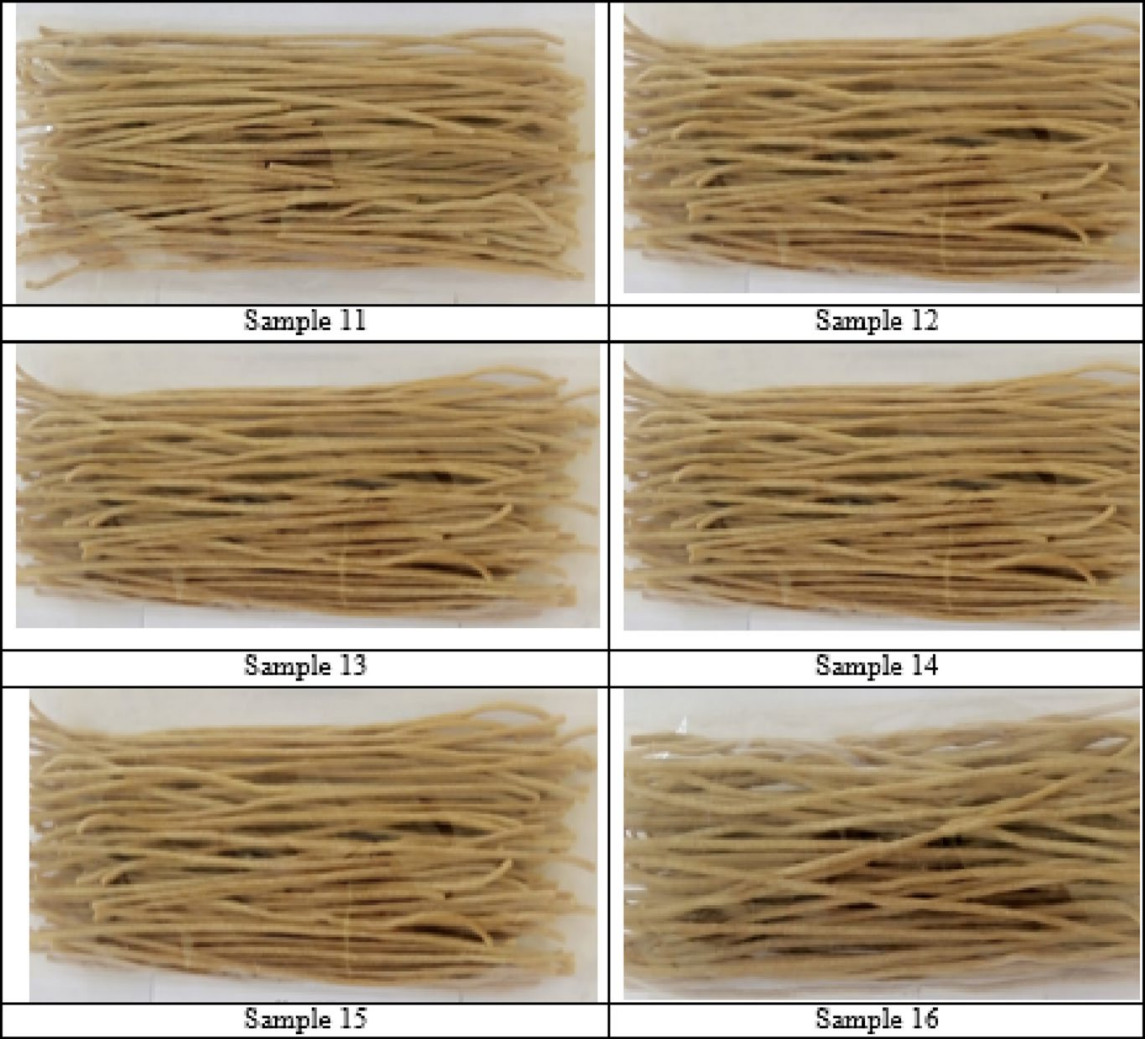
Samples of pasta made of whole grain durum wheat ‘Satti’ flour, carrot powder and ion-ozonated water.

In order to develop a range of pastas based on the durum wheat ‘Satti’ flour, carrot powder and ion-ozonated water, 16 samples were prepared featuring different concentrations of carrot powder (C_cp._, x_3_) and ozone in the water (C_io_, x_1_), and different mixing water temperatures (t_w_, x_2_) and drying temperatures (t_d_, x_4_) (Fig. 1). We studied the dependence of pasta quality indicators on multiple parameters of pasta production, including the C_io_ (C_io_ × 10^− 6^, mg/cm^3^), the t_w_ used in kneading the pasta dough (°C), the C_cp._ (%) and the t_d_ of the pasta (°C). From the made pasta products, we determined the moisture content (%, y_1_), acidity (degree, y_2_), preservation of the shape (%, y_3_), coefficient of the increase in mass (C_m_, y_4_), coefficient of the increase in the volume (C_v_, y_5_), number of DM transferred to the cooking water (%, y_6_), cooking time until ready (min, y_7_), complete deformation (H_1_, mm, y_8_), plastic deformation (H_2_, mm, y_9_), elastic deformation (H_3_, mm, y_10_), protein content (%, y_11_), starch content (%, y_12_), carbohydrate content (%, y_13_), fat content (%, y_14_), fibre content (%, y_15_), ash content (%, y_16_), vitamin A content (mg/100 g, y_17_), vitamin E content (mg/100 g, y_18_), vitamin C content (mg/100 g, y_19_), β-carotene content (mg/100 g, y_20_), Ca content (mg/100 g, y_21_), K content (mg/100 g, y_22_), Mg content (mg/100 g, y_23_), Fe content (mg/100 g, y_24_) and zinc (Zn) content (mg/100 g, y_25_).

**Fig. 2:**
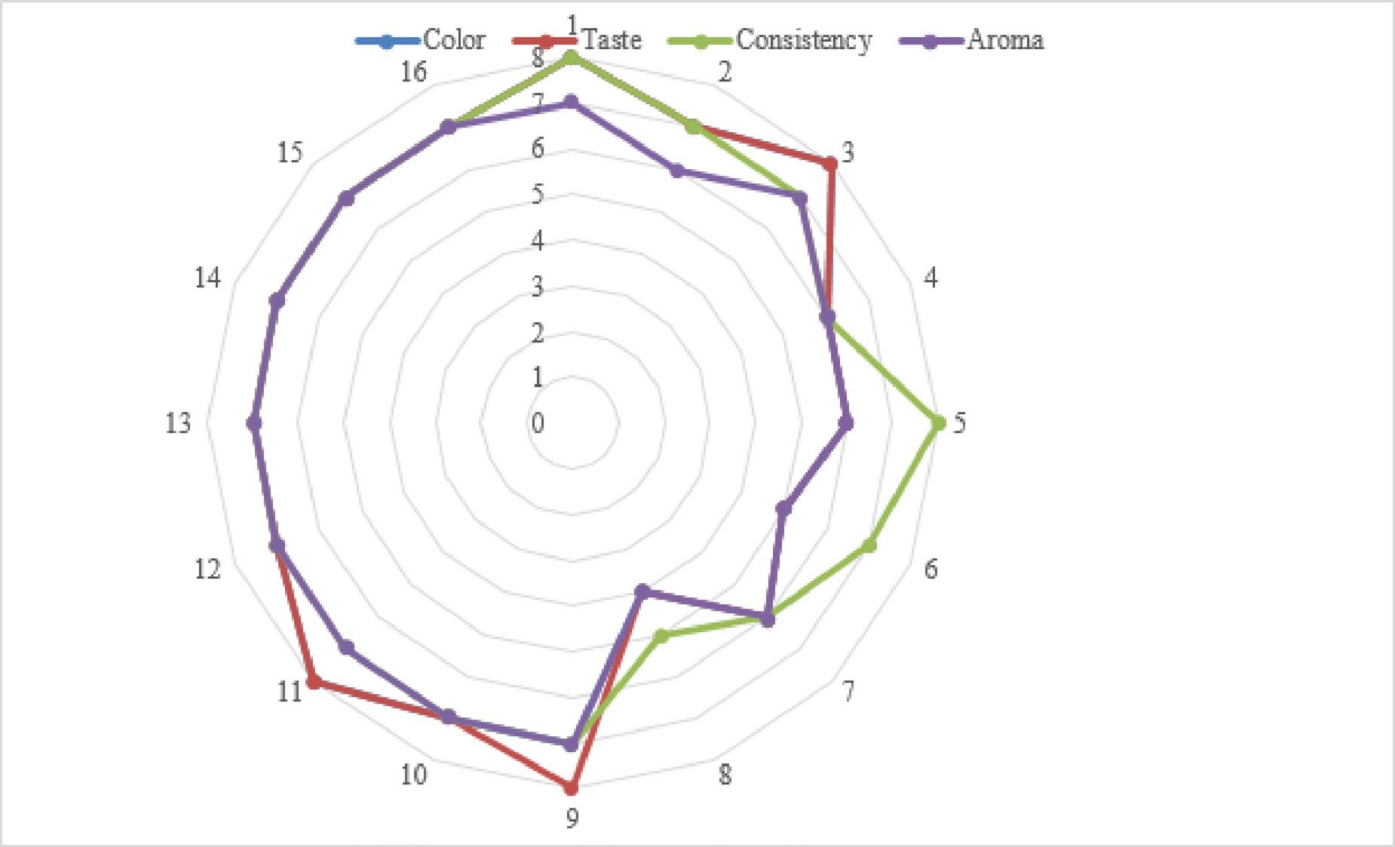
Organoleptic characteristics of the 16 pasta samples.

The 16 pasta samples were subjected to a sensory evaluation based on five main indicators––colour, taste, aroma, consistency and overall organoleptic appeal. Each sample received an integral point score on a 9-point scale (Fig. 2).

Samples 1–8 (dried at 60 °C) had moderate sensory characteristics. The best results were recorded for the samples with a higher C_io_ (2.5 mg/cm³), especially mixed at a t_w_ of 60 °C and with the addition of 3% carrot powder (samples 1 and 3). These were distinguished by a rich colour, pronounced taste and elastic consistency. By contrast, the samples with a C_io_ of 1.5 mg/cm³ (samples 2, 4, 6, 8) had a less pronounced aroma and colour, which we associated with a lower water activity during mixing. The samples with 1% carrot powder generally had a weaker vegetable note in taste and colour.

Samples 9–16 (dried at 50 °C) had the highest and most balanced sensory characteristics, especially at a C_io_ of 2.5 mg/cm³ and a C_cp._ of 3% (samples 9 and 11). These had a bright natural colour, pleasant aroma and harmonious taste, while maintaining a dense and elastic consistency. Using a lower t_d_ (50 °C) had a positive effect on the preservation of the natural colour and flavour, which was especially notable in those samples with the addition of carrot powder. In addition, a high C_io_ contributed to the formation of a more uniform texture and enhanced the freshness of the aroma. Samples 10, 12, 14 and 16, despite having a C_io_ of 1.5 mg/cm³, also demonstrated acceptable sensory indicators after adjusting the process parameters. This shows the potential for improvement even at lower ozone doses, provided other factors (e.g. t_d_ and C_cp._) are optimised.

We found that the following factors had the greatest influence on the sensory qualities of the pasta: a C_io_ of 2.5 mg/cm³ gave the best effect in terms of taste, colour and texture; a mixing t_w_ of 60 °C ensured more uniform hydration; a t_d_ of 50 °C helped preserve the aroma and colour; and a C_cp._ of 3% was optimal in terms of taste, colour and aroma, with 1% having an insufficient effect.

The sensory analysis confirmed that the pasta samples with 3% carrot powder, 2.5 mg/cm³ C_io_ and a t_d_ of 50 °C (samples 9 and 11) were the most successful in terms of their organoleptic properties, confirming the potential to achieve high product quality without compromising on its naturalness and nutritional value.

To reliably assess the influence of individual factors on the quality of the pasta, we applied a multifactorial experimental planning method using the sequential regression analysis program PLAN^34,35^. To reduce the influence of uncontrolled factors on the experimental results, the experiments were randomised using tables of random numbers. The experimental conditions and quality indicators of the pasta samples are shown in Tables 2, 3 and 4.

**Table 2:** Experimental conditions and physicochemical quality indicators of the pasta samples.

NSample no.	Factor				Physicochemical quality indicator						
	C_io_ (C_io_ ×10^6^, Mg/cm^3^)	t_w_ (°C)	C_cp._ (%)	t_d_ (°C)	Humidity (%)	Acidity (degree)	Shape preservation (%)	Coefficient of mass increase (C_m_)	Coefficient of volume increase (C_v_)	DM transferred to cooking water (%)	Cooking time until ready (min)
	х_1_	х_2_	х_3_	х_4_	у_1_	у_2_	у_3_	у_4_	у_5_	у_6_	у_7_
1	2.5	60	3.0	60	12.6	4.0	98	1.97	1.10	6.00	15
2	1.5	60	3.0	60	12.2	3.8	95	2.18	1.23	6.40	13
3	2.5	50	3.0	60	12.4	3.6	100	2.01	1.10	5.98	15
4	1.5	50	3.0	60	12.6	3.6	95	2.23	1.21	6.41	14
5	2.5	60	1.0	60	12.4	3.62	100	2.04	1.10	5.98	15
6	1.5	60	1.0	60	12.6	3.6	100	2.03	1.10	5.95	15
7	2.5	50	1.0	60	12.6	3.6	100	2.01	1.10	5.94	15
8	1.5	50	1.0	60	12.6	4.0	100	1.98	1.10	5.94	15
9	2.5	60	3.0	50	12.8	3.4	100	1.82	1.02	5.81	15
10	1.5	60	3.0	50	12.8	3.5	100	1.92	1.04	5.87	15
11	2.5	50	3.0	50	13.0	3.6	100	1.78	1.02	5.76	16
12	1.5	50	3.0	50	13.0	3.6	100	1.90	1.01	5.84	15
13	2.5	60	1.0	50	13.0	3.6	100	1.98	1.10	5.95	15
14	1.5	60	1.0	50	13.0	3.8	96	2.14	1.10	6.41	13
15	2.5	50	1.0	50	13.0	3.4	100	2.06	1.22	6.00	15
16	1.5	50	1.0	50	13.0	3.6	100	1.99	1.10	5.89	15

**Table 3:** Rheological quality indicators of the pasta samples.

Sample no.	Total deformation (H_1_, mm)	Plastic deformation (H_2_, mm)	Elastic deformation (H_3_, mm)
	у_8_	у_9_	у_10_
1	4.59	6.11	1.15
2	3.70	3.09	0.84
3	4.05	2.69	0.86
4	3.34	2.33	1.01
5	1.62	3.02	0.71
6	1.63	1.79	0.61
7	1.91	3.14	0.98
8	1.58	1.73	0.70
9	1.48	4.08	0.72
10	1.34	3.56	0.61
11	1.74	2.54	0.75
12	2.31	2.04	1.02
13	3.47	2.33	0.85
14	3.91	1.51	0.98
15	4.27	5.45	1.03
16	3.22	1.96	0.62

The experimental conditions correspond to Table 2.

**Table 4:** Сhemical composition of the pasta samples.

Sample no.	Protein (%)	Starch (%)	Carbohydrate (%)	Fat (%)	Fibre (%)	Ash (%)
	у_11_	у_12_	у_13_	у_14_	у_15_	у_16_
1	13.20	62.12	67.52	2.75	6.07	1.46
2	13.24	62.56	67.58	2.70	6.09	1.41
3	13.22	61.97	67.55	2.73	6.13	1.54
4	13.29	61.56	67.45	2.64	6.08	1.53
5	14.07	67.42	73.28	2.85	3.71	1.09
6	14.09	67.58	73.44	2.83	3.69	1.14
7	14.13	66.98	73.23	2.75	3.68	1.18
8	14.10	66.99	73.28	2.82	3.74	1.12
9	13.19	61.95	67.38	2.78	6.18	1.54
10	13.28	61.98	67.23	2.69	6.04	1.57
11	14.09	67.55	73.48	2.84	3.84	1.46
12	13.25	62.59	67.25	2.74	6.07	1.58
13	14.18	66.78	73.18	2.81	3.83	1.07
14	14.25	66.59	73.11	2.79	3.78	1.13
15	14.28	66.35	73.07	2.65	3.81	1.12
16	14.24	66.48	73.12	2.68	3.80	1.09

The experimental conditions correspond to Table 2.

The acidity of pasta is largely influenced by the acidity of the initial flour. Based on the data in Table 4, the acidity levels of the pasta samples ranged between 3.4 and 4.0 degrees, aligning with standard requirements. As shown in Table 3, the cooking time for the pasta samples varied between 13 and 16 min. The mass increase coefficient ranged from 1.78 to 2.23. Closely associated with these parameters is a key indicator of pasta cooking properties—the amount of DM released into the cooking water. This value fluctuated between 5.76% and 6.41%.

The quality assessment of the pasta samples revealed that samples 9–12 exhibited superior quality indicators compared to the others. Conversely, samples 2, 4, and 14 had lower quality characteristics. The lowest DM loss into the cooking water (5.76%) was recorded for sample 11, where the influencing factors were x_1_ = 2.5 × 10^−6^ mg/cm^2^, x_2_ = 50 °C, x_3_ = 3.0% and x_4_ = 50 °C. The highest protein content (14.28%) was observed in sample 15, with the influencing factors x_1_ = 2.5 × 10^−6^ mg/cm^2^, x_2_ = 50 °C, x_3_ = 1.0% and x_4_ = 50 °C (Tables 4 and 5).

**Table 5:** Vitamin and mineral composition of the pasta samples.

Sample no.	Vitamins (mg/100 g)				Mineral substances (mg /100 g)					
	А	Е	С	β-carotene	Са	K	Mg	Fe	Zn	
	у_17_	у_18_	у_19_	у_20_	у_21_	у_22_	у_23_	у_24_	у_25_	
1	1.44	3.85	2.76	11.04	88.21	398.52	32.95	1.67	0.92	
2	1.43	3.78	2.74	11.02	89.32	386.53	32.13	1.53	0.87	
3	1.37	3.65	2.72	11.07	87.93	379.33	31.47	1.62	0.91	
4	1.34	3.74	2.78	11.06	88.18	391.35	29.83	1.60	0.89	
5	0.37	2.13	0.92	3.68	87.38	258.16	27.65	1.36	0.78	
6	0.42	2.18	0.92	3.64	85.73	250.16	27.61	1.40	0.77	
7	0.39	2.22	1.10	3.59	86.12	251.73	26.93	1.37	0.73	
8	0.38	2.19	0.98	3.61	83.49	249.18	26.39	1.39	0.78	
9	1.39	3.68	2.74	11.08	89.12	368.17	28.09	1.60	0.75	
10	1.35	3.72	2.73	11.06	90.01	373.01	28.17	1.55	0.77	
11	1.33	3.53	2.63	10.54	83.41	350.81	27.93	1.54	0.75	
12	1.37	3.71	2.71	11.07	91.13	380.00	26.13	1.47	0.73	
13	0.44	2.14	0.94	3.72	83.48	251.10	27.85	1.28	0.74	
14	0.43	2.18	0.97	3.71	83.55	250.92	27.69	1.31	0.75	
15	0.39	2.13	0.93	3.67	83.58	250.84	27.74	1.26	0.76	
16	0.41	2.10	0.91	3.68	83.61	250.88	27.78	1.23	0.78	

The experimental conditions correspond to Table 2.

The quality of pasta products is largely influenced by the rheological characteristics of the dough. Throughout the technological process, these properties undergo continuous changes due to various factors, including transformations in the state and structure of the raw material components, shifts in the ratio of liquid to solid phases and the activity of the dough enzymes. Processing involves complex physicochemical and mechanical interactions. Flour-based dough represents a heterogeneous colloidal dispersed system, where the addition of different improvers can modify its rheological behaviour, enhancing its consistency and relaxation properties. To evaluate the impact of finely dispersed plant-based additives and ion-ozonated water on the rheological characteristics (total, plastic and elastic deformation) of the doughs the 16 pasta samples were made from, changes in the doughs’ physical properties were analysed using a structurometer.

Investigation of the plastic and elastic deformation of the doughs showed that the quality (physical properties) of finished pasta samples 9–12 was better than the other samples, with the lowest values being recorded in dough samples 2, 4 and 14.

The regression coefficients were calculated using matrices in the natural dimension. Accordingly, the equations themselves were also obtained in the natural dimension. The general form of the regression equations for four factors is as follows:

1 $$\begin{array}{ccccc}{{\rm{y}}_{\rm{i}}} & = {\rm{b}}0 + {\rm{b1}}{_{{\rm{i}}}} + {\rm{b2}}{{\rm{t}}_{\rm{w}}}\\& \quad + {\rm{b3}}{{\rm{C}}_{{\rm{cp}}}} + {\rm{b4 }}{{\rm{t}}_{\rm{d}}} + {\rm{b12}}{_{{\rm{i}}}}{{\rm{t}}_{\rm{w}}}\\& \quad + {\rm{b13}}{_{{\rm{i}}}}{{\rm{C}}_{{\rm{cp}}}} + {\rm{b14}}{_{{\rm{i}}}}{{\rm{t}}_{\rm{d}}} + {\rm{b23}}{{\rm{t}}_{\rm{w}}}{{\rm{C}}_{{\rm{cp}}}}\\& \quad + {\rm{b24}}{{\rm{t}}_{\rm{w}}}{{\rm{t}}_{\rm{d}}} + {\rm{b34}}{{\rm{C}}_{{\rm{cp}}}}{{\rm{t}}_{\rm{d}}}\end{array}$$

where y_i_ is the i-th indicator of the quality of the dough and the pasta made from it.

The experimental conditions and chemical compositions of the pasta samples are shown in Tables 4 and 5.

Analysing the results of the elemental composition of the pasta samples, we found that the pasta augmented with carrot powder contained higher levels of essential minerals, such as Mg, Fe, Ca, K and Zn. In particular, the pasta enriched with carrot powder had an increased Fe content (1.25–1.72 mg) compared to the control (1.06 mg) and increased Ca levels (83.12 compared to 91.13 mg). Augmentation with carrot powder also improved the vitamin content of the pasta. Thus, even a small amount of carrot powder proved to be beneficial, enhancing the nutritional value of the pasta by enriching it with essential food components.

The obtained regression equations made it possible to predict the quality indicators of the pasta based on the parameters (i.e., the failure factors, C_io_, t_w_, С_cp._ and t_d_). The parameters C_io_, t_w_, C_cp._ and t_d_ are shown in Table 6. The standard deviations (Se and Si.ad.) and the Fisher’s criteria (F_c_ and F_cr_), indicating that all the equations adequately describe the experimental data with a confidence probability of *p* = 0.05, are included in Table 6.

**Table 6:** Regression equations for the natural variables And statistical characteristics of the dependences of the quality indicators of the pasta samples on the factors affecting them (C_io_, t_w_, C_cp._ And t_d_).

Regression equations for natural variables	Standard deviation		Fisher criterion	
	Experimental (S_e_)	Inadequacies (S_i.аd_.)	Calculated (F_c_)	Critical (F_cr_)
y_1_ = 12.725	0.262	0.231	1.29	19.43
y_2_ = 3.657	0.180	0.178	1.02	3.68
y_3_ = 99.00	2.900	1.897	2.34	3.68
y_4_ = 2.055–0.4112 × C_cp._ + 0.00700·C_cp._ × t_d_	0.052	0.088	2.88	19.42
y_5_ = 1.102	0.120	0.066	3.30	3.68
y_6_ = 6.007–0.5617 × C_cp._ + 0.01022 × C_cp._ × t_d_	0.102	0.185	3.31	19.42
y_7_ = 14.750–0.7000 × C_cp._ + 0.3500 × С_iо_ × 10^–6^ × C_cp._	0.290	0.710	5.99	19.42
y_8_ = 25.467–11.59 × C_cp._ – 0.41500 × t_d_ + 0.2117 × C_cp._·t_d_	0.712	0.420	2.87	3.89
y_9_ = 11.90 + 1.419 × С_iо_ × 10^–6^ – 0.2266 × t_w_ – 7.129 × C_cp._ + 0.1359 × t_w_ × C_cp._	0.340	0.827	5.91	19.40
y_10_ = 2.292–0.8225 × C_cp._ – 0.02750 × t_d_ + 0.01550 × C_cp._ × t_d_	0.041	0.166	16.41	19.41
y_11_ = 15.55–0.4112 × C_cp._ – 0.01775 × t_d_	0.081	0.208	6.60	19.42
y_12_ = 68.95–2.056 × C_cp._	1.240	1.416	1.30	19.42
y_13_ = 75.73–2.517 × C_cp._	1.530	1.519	1.01	3.74
y_14_ = 2.753	0.120	0.068	3.13	3.68
y_15_ = 2.603 + 1.152 × C_cp._	0.131	0.087	2.24	3.74
y_16_ = 0.9206 + 0.1969 × C_cp._	0.043	0.049	1.31	19.42
y_17_ = − 0.08312 + 0.4869 × C_cp._	0.031	0.033	1.17	19.42
y_18_ = 1.384 + 0.7744 × C_cp._	0.132	0.072	3.32	3.74
y_19_ = 0.91375 × C_cp._	0.034	0.063	3.41	19.43
y_20_ = 3.664 × C_cp._	0.131	0.129	1.02	3.68
y_21_ = 82.72 + 1.89812 × C_cp._	1.260	1.955	2.41	19.42
y_22_ = 188.20 + 63.42 × C_cp._	15.330	10.656	2.07	3.74
y_23_ = 26.39–5.217 × C_cp._ + 0.1142 × C_cp._ × t_d_	0.820	1.112	1.84	19.42
y_24_ = 1.242 + 0.05180 × С_iо_ × 10^–6^ × C_cp._	0.071	0.074	1.08	19.42
y_25_ = 0.7300–0.2162 × C_cp._ + 0.00450 × C_cp._ × t_d_	0.036	0.025	2.11	3.81

Analysis of the regression equations in Table 6 showed that indicators of pasta quality, such as product moisture (y_1_), acidity (y_2_), shape preservation (y_3_), product volume increase coefficient (y_5_) and fat content (y_14_), do not depend on technological parameters. Indicators of pasta quality dependent on one factor, C_cp._, were starch (y_12_), carbohydrates (y_13_), fibre (y_15_), ash (y_16_), vitamins (A (y_17_), E (y_18_), C (y_19_) and β-carotene (y_20_)), Ca (y_21_) and K (y_22_).

Only the duration of cooking until ready (y_7_) depended on two regime factors—C_io_ and C_cp._. The C_cp._ and t_d_ depended on quality indicators such as the coefficient of increase in product mass (y_4_,), the amount of DM transferred to the cooking water (y_6_), the total deformation (H_1_, y_8_), elastic deformation (H_3_, y_10_), protein content (y_11_) and mineral content (i.e. Mg (y_23_) and Zn (y_25_)). Only plastic deformation (H_2_, y_9_) depended on the three-mode factors of C_io_, C_cp._ and t_w_. None of the indicators of pasta quality depended on all four factors.

To optimise the parameters of carrot-augmented pasta production, the following quality indicators were selected as target functions:

Protein content (%):

2 $${{\rm{y}}_{{\rm{11}}}} = {\rm{ 15}}.{\rm{55}}{-}0.{\rm{4112}} \times {{\rm{C}}_{{\rm{cp}}}}{-}0.0{\rm{1775}} \times {\rm{ }}{{\rm{t}}_{\rm{d}}} \to {\rm{ max}}$$

Amount of DM transferred to the cooking water (%):

3 $${{\rm{y}}_{\rm{6}}} = {\rm{6}}.00{\rm{7}}{-}0.{\rm{5617}} \times {{\rm{C}}_{{\rm{cp}}}} + 0.0{\rm{1}}0{\rm{22}} \times {{\rm{C}}_{{\rm{cp}}}} \times {\rm{ }}{{\rm{t}}_{\rm{d}}} \to {\rm{ }}\min$$

**Fig. 3:**
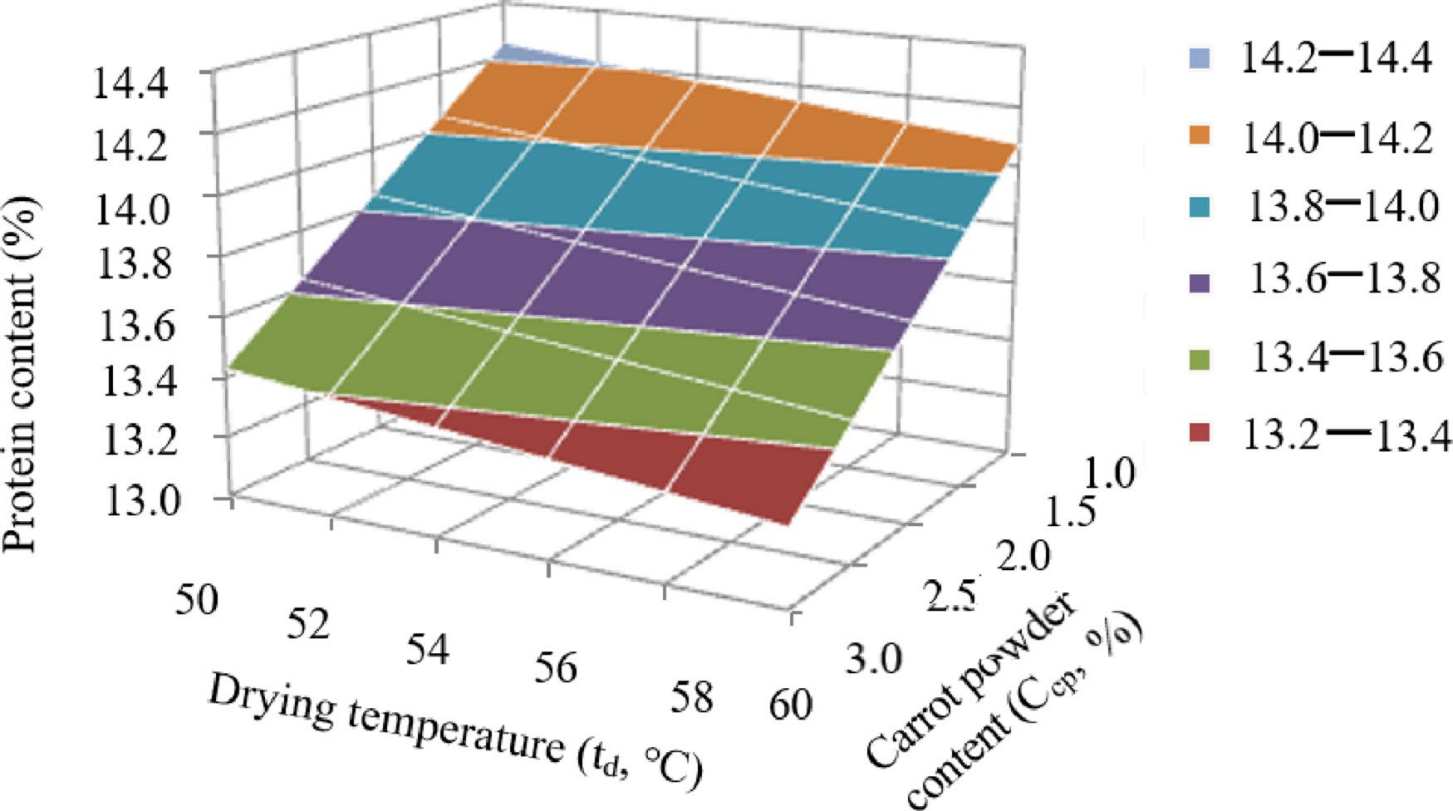
Response surface for the influence of C_cp._ and t_d_ of the pasta on the protein content of the pasta.

An analysis of Eq. (2) shows that only two regime factors affected the protein content—C_cp._ and t_d_. Due to the insignificance (absence) of the coefficient of the pair interaction, the nature of the influence of these factors was linearly inversely proportional, as clearly visible from the response surface (Fig. 3) constructed based on Eq. (2).

Figure 3 shows that increasing the t_d_ of the pasta, regardless of the C_cp._, decreases the protein content by 0.18%. Increasing the C_cp._, regardless of the t_d_ of the pasta, leads to a more significant decrease in protein content of 0.82%. We concluded that, in the range of differences in C_cp._ and t_d_ of the pasta, introducing the carrot powder had a 4.63 times stronger effect on the protein content. Thus, to increase the protein content of the pasta, the C_cp._ should be reduced and the pasta dried at a lower temperature. Figure 3 clearly shows that the maximum protein content (14.25%) is achieved by including 1.0% of carrot powder and drying the pasta at 50 °C.

**Fig. 4:**
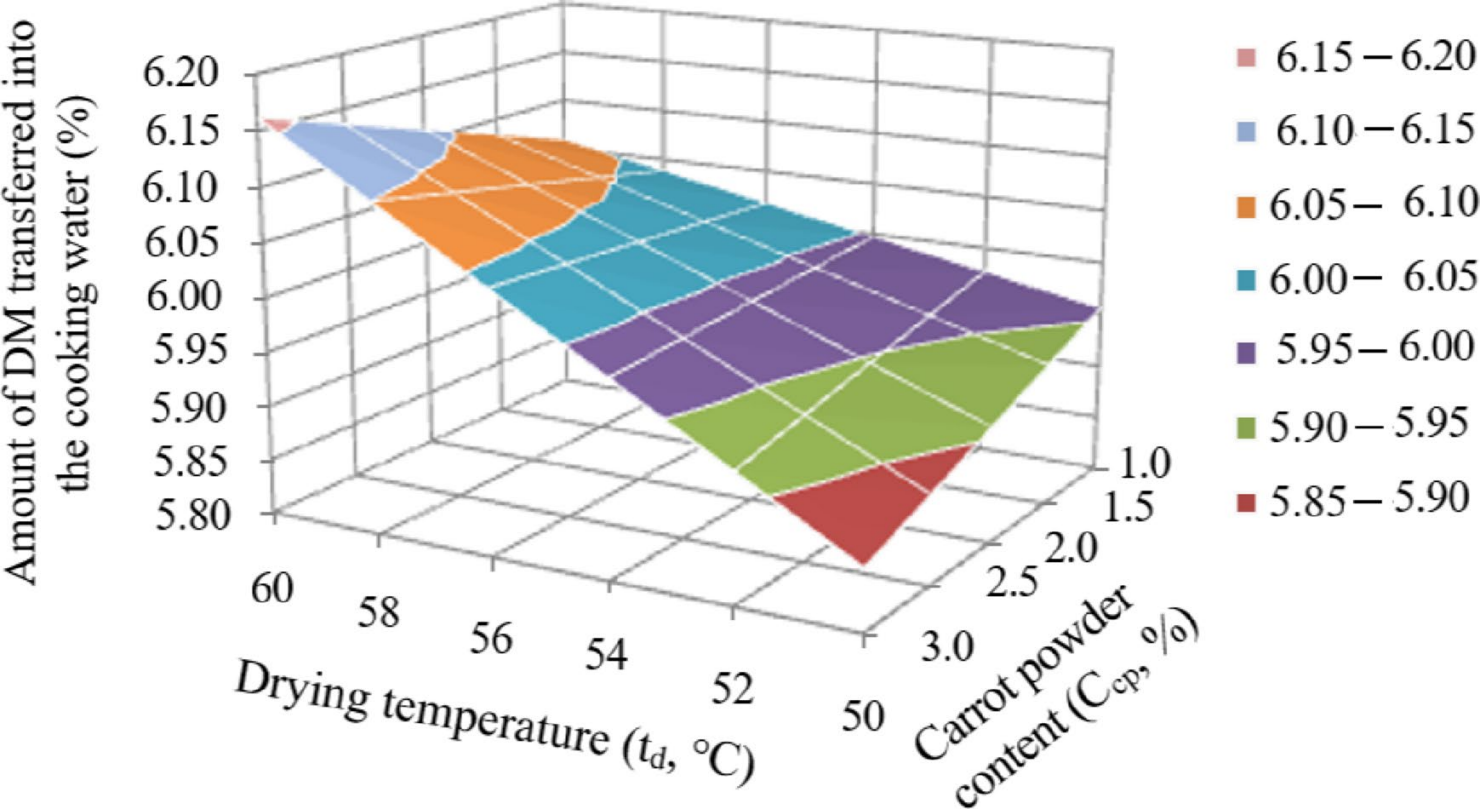
Surface of the response of the joint interaction of the factors C_cp._ and t_d_ on the amount of DM transferred to the cooking water.

As noted above, the second target function was the amount of DM passing into the cooking water, which, as with the protein content, depended on C_cp._ and t_d_. Analysis of Eq. (3) showed that only two regime factors affected DM loss—C_cp._ and t_d_—and this influence manifested itself in the form of a paired contradictory interaction of these factors (the coefficient of paired interaction is 0.01022 × C_cp._ × t_d_). Because of this, according to the equation for the natural variables, it was difficult to determine the effect of each individual factor on the protein content. The nature of the joint interaction of C_cp._ and t_d_ on the DM content (y_6_) passing into water can be clearly seen from the response surface (Fig. 4), based on Eq. (3).

Analysis of the reduced response surface showed that any increase in t_d_, regardless of the C_cp._ introduced, increased the loss of DM into the cooking water. The effect of the introduced carrot powder significantly depended on the t_d_. Therefore, at t_d_ = 50 °C, an increase in C_cp._ reduces the loss of DM into the cooking water. By contrast, at t_d_ = 60 °C, the transition of DM into the cooking water decreases with an increase in C_cp._. Thus, in order to reduce the loss of DM into the cooking water, it is necessary to increase the C_cp._ introduced into the pasta and reduce the t_d_.

Table 7 presents the results of the regression analysis, including the values of the coefficients, their standard errors (Std. Err.), t statistics, significance levels (p values) and 95% confidence intervals.

**Table 7:** Regression coefficients.

Coefficient	Coef.	Std.Err.	t	*p* > |t|	[0.025	0.975]
C_cp._	0.40750	0.00382	106.70855	0.00001	0.39816	0.41684
t_d_	0.21704	0.00014	1525.03904	0.00001	0.21669	0.21739

From the data in Table 7, it can be seen that the coefficient of C_cp._ is 0.4075, which means that an increase in the C_cp._ of 1% would increases the protein content in the pasta by an average of 0.41%, all other things being equal.

The coefficient of t_d_ = 0.21704, which indicates a direct relationship—an increase in t_d_ of 1 °C increases the protein content by 0.22%. For both variables, *p* = 0.00001, which is significantly lower than the significance level of 0.05. This indicates that both factors had a highly significant effect on the target indicator (protein content). The low standard errors (0.00382 for C_cp._ and 0.00014 for t_d_) indicated high accuracy of the coefficient estimates. The t statistic values were extremely high (> 100 and > 1500), which confirmed the reliability of the model and the absence of randomness in the estimates obtained. The confidence interval (95%) for C_cp._ was [0.39816, 0.41684] and for t_d_, [0.21669, 0.21739]. These narrow confidence intervals further confirmed the stability of the model and the low dispersion of the estimates. The resulting regression model is highly reliable and statistically significant. Both included factors—C_cp._ and t_d_—reliably and directly affected the protein content in the finished product. The narrow confidence intervals and low standard errors indicate high forecast accuracy, which allows us to recommend this model for use in optimising the process modes in producing the pasta.

Optimisation of the pasta production process based on the selected optimality functions was carried out with consideration of the constraints on the quality indicators of the finished product. The ranges of the process variables—C_io_, tw, C_cp._, and t_d_—were determined according to the experimental design matrix. A nonlinear programming method was applied for the optimisation. The values of the remaining quality indicators for the carrot-powder-enriched pasta obtained under conditions ensuring optimal protein content (y_11_) and minimal loss of dry matter (DM) in the cooking water (y₆) are presented in Table 8.

**Table 8:** Restrictions on pasta quality indicators and quality indicators of the pasta obtained under conditions ensuring an optimal protein content (y_11_) and minimal loss of DM in the cooking water (y_6_).

Min		Pasta quality indicators and their equations	Optimum			Max
			у_11_	у_6_		
12.0	≤	pasta humidity (%): y_1_ = 12.725	12.73	12.73	≤	14.0
3.0	≤	pasta acidity (degree): y_2_ = 3.657	3.66	3.66	≤	4.5
82.0	≤	preservation of pasta shape (%): y_3_ = 99.00	99.00	99.00	≤	100.0
2.1	≤	coefficient of increase in product mass (C_m_): y_4_ = 2.055 – 0.4112× C_cp._ + 0.00700 × C_cp._ × t_d_	1.99	1.87	≤	2.55
1.19	≤	coefficient of increase in product volume (C_v_): y_5_ = 1.102	1.10	1.10	≤	1.63
5.8	≤	amount of DM transferred to the cooking water (%): y_6_ = 6.007–0.5617 × C_cp._ + 0.01022 × C_cp._ × t_d_	5.95	5.86	≤	7.2
10.0	≤	cooking time until ready (min): y_7_ = 14.750–0.7000 × C_cp._ + 0.3500 × С_iо_ × 10^–6^ × C_cp._	4.57	14.22	≤	15.5
1.0	≤	total deformation, H_1_ (mm): y_8_ = 25.467–11.59 × C_cp._ – 0.41500 × t_d_ + 0.2117 × C_cp._ × t_d_	3.72	1.72	≤	3.0
0.4	≤	plastic deformation, H_2_ (mm): y_9_ = 11.90 + 1.419 × С_iо_ × 10^–6^ – 0.2266 × t_w_ – 7.129 × C_cp._ + 0.1359 × t_w_ × C_cp._	2.36	1.69	≤	2.25
0.58	≤	elastic deformation, H_3_ (mm): y_10_ = 2.292–0.8225 × C_cp._ – 0.02750 × t_d_ + 0.01550 × C_cp._ × t_d_	0.87	0.78	≤	2.2
12.55	≤	protein content (%): y_11_ = 15.55–0.4112 × C_cp._ – 0.01775 × t_d_	14.26	13.43	≤	14.6
65.0	≤	starch content (%): y_12_ = 68.95–2.056 × C_cp._	66.90	62.79	≤	69.9
70.0	≤	carbon content (%): y_13_ = 75.73–2.517 ×C_cp._	73.21	68.18	≤	76.0
2.45	≤	fat content (%): y_14_ = 2.753	2.75	2.75	≤	3.6
1.1	≤	fibre content (%): y_15_ = 2.603 + 1.152 × C_cp._	3.76	6.06	≤	3.8
2.0	≤	ash content (%): y_16_ = 0.9206 + 0.1969 × C_cp._	1.12	1.51	≤	4.8
0.3	≤	vitamin A content (mg/100 g): y_17_ = − 0.08312 + 0.4869 × C_cp._	0.40	1.38	≤	1.5
3.45	≤	vitamin E content (mg/100 g): y_18_ = 1.384 + 0.7744 × C_cp._	2.16	3.71	≤	8.55
1.4	≤	vitamin C content (mg/100 g): y_19_ = 0.91375 × C_cp._	0.91	2.74	≤	4.9
5.4	≤	β-carotene content (mg/100 g): y_20_ = 3.664 × C_cp._	3.66	10.99	≤	19.5
117.85	≤	Ca content (mg/100 g): y_21_ = 82.72 + 1.89812 × C_cp._	84.62	88.41	≤	197.6
447.0	≤	K content (mg/100 g): y_22_ = 188.20 + 63.42 × C_cp._	251.62	378.67	≤	561.5
45.1	≤	Mg content (mg/100 g): y_23_ = 26.39–5.217 × C_cp._ + 0.1142 × C_cp._ × t_d_	26.88	27.87	≤	96.35
1.65	≤	Fe content (mg/100 g): y_24_ = 1.242 + 0.05180 × С_iо_ × 10^–6^ × C_cp._	1.32	1.47	≤	3.25
0.5	≤	Zn content (mg/100 g): y_25_ = 0.7300–0.2162 × C_cp._ + 0.00450 × C_cp._ × t_d_	0.74	0.76	≤	1.95

Table 8 shows that the maximum amount of protein in the finished pasta (14.25%) was provided by the optimal parameters C_cp._ = 1.0% and t_d_ = 50 °C. The C_io_ and t_w_ did not affect the protein content. The minimum amount of DM transferred to the cooking water (5.86%) was ensured by the optimal parameters C_cp._ = 3.0% and t_d_ = 50 °C. The C_io_ and t_w_ did not affect the amount of DM transferred to the cooking water.

The optimisation of the pasta production process based on the selected optimality functions (y_11_ and y_6_) yielded opposite optimal conditions: C_cp._ = 1.0% for maximum protein content and 3.0% for minimum dry matter (DM) loss during cooking. Consequently, one of these functions must be prioritised, while the other serves as a constraint on the corresponding quality parameter. Alternatively, a compromise solution can be found using a generalised quality criterion calculated from y_11_ and y_6_.

Enrichment of pasta with functional ingredients is a growing area of food research aimed at improving nutritional value and health outcomes. Many studies highlight the importance of using vegetable powders, fibres, or bioactive compounds to enhance pasta functionality while maintaining acceptable texture and cooking quality^36,37^. To assess the influence of each factor on pasta quality, a multifactorial experimental design with sequential regression analysis (PLAN software) was applied^38,39^. This approach enabled the identification of statistically significant regression coefficients and verification of model adequacy for each quality indicator.

Quantitative assessment of pasta quality showed that the minimum DM loss (5.76%) occurred under conditions of C_io_ = 2.5 × 10^−6^ mg/cm^2^, tw = 50 °C, C_cp._ = 3.0%, and t_d_ = 50 °C. These values fall below the GOST 31743-2017 threshold (≤ 6.0%), confirming high structural stability and shape retention during cooking. Compared with similar studies, our results demonstrated lower DM loss: spinach-enriched pasta exhibited 6.1–6.3% loss, while pumpkin-enriched samples showed 6.0–6.5%. Thus, the use of carrot powder under gentle drying conditions improved technological performance, likely due to its fibre content and structural-binding effect.

Although the effect of ion-ozonated water on protein and DM loss was statistically insignificant, its inclusion in the process remains justified. Ion-ozonated water contributed to improved microbiological stability and hygienic safety, enabling the production of pasta under “clean label” conditions without enzyme or stabiliser additives^40,41^. Similar approaches have been proposed for extending shelf life and improving microbial safety in minimally processed foods. Therefore, its application adds technological and safety value, even if its direct physicochemical effect is limited^42–44^.

The maximum protein content (14.28%) was achieved at C_io_ = 2.5 × 10^−6^ mg/cm^2^, t_w_ = 50 °C, C_cp._ = 1.0%, and t_d_ = 50 °C. This value is comparatively high—similar studies using carrot or spinach powder report protein levels of 13.2–13.6%, often achieved by adding gluten or soy protein concentrates. In our case, the high protein content was obtained without additional protein fortifiers, suggesting a well-balanced product matrix. Regression analysis indicated that C_cp._ and td significantly affected protein content, while C_io_ and t_w_ did not, consistent with findings by Gökmen^45^, Ingvarsson et al.^46^ and Fazullina et al.^47^, who emphasised protein stabilisation during low-temperature drying.

An increase in carrot powder content from 1.0% to 3.0% resulted in a decrease in protein content, consistent with the dilution effect reported in other studies on vegetable-enriched pasta. Similar trends were observed by Beşir et al.^48^, Gökmen et al.^49^ and Mounika et al.^50^, where increasing vegetable powder inclusion reduced relative protein concentration due to replacement of wheat flour. However, this same increase improved DM retention, highlighting the trade-off between nutritional and technological optimisation targets.

Nonlinear programming methods were used to identify process conditions that balanced minimal DM loss and maximal protein content. Optimal parameters for reducing DM loss were Ccp = 3.0% and td = 50 °C (DM loss = 5.86%), while maximum protein content was achieved at Ccp = 1.0% and td = 50 °C (protein = 14.25%). These findings confirm the need for compromise optimisation to balance nutritional quality and technological stability.

Overall, the use of carrot powder and ion-ozonated water produced pasta with desirable technological and nutritional characteristics. The inclusion of carrot powder enriched the product with β-carotene and fibre, improving both health value and structural integrity. Although ion-ozonated water did not significantly alter protein or DM loss, its benefits in microbiological control and hygienic processing justify its continued use. The developed recipe and processing approach demonstrate a scientifically sound and safe technology for producing high-quality, functional pasta with reduced additive dependency and enhanced nutritional profile.

## Materials and methods

**Fig. 5:**
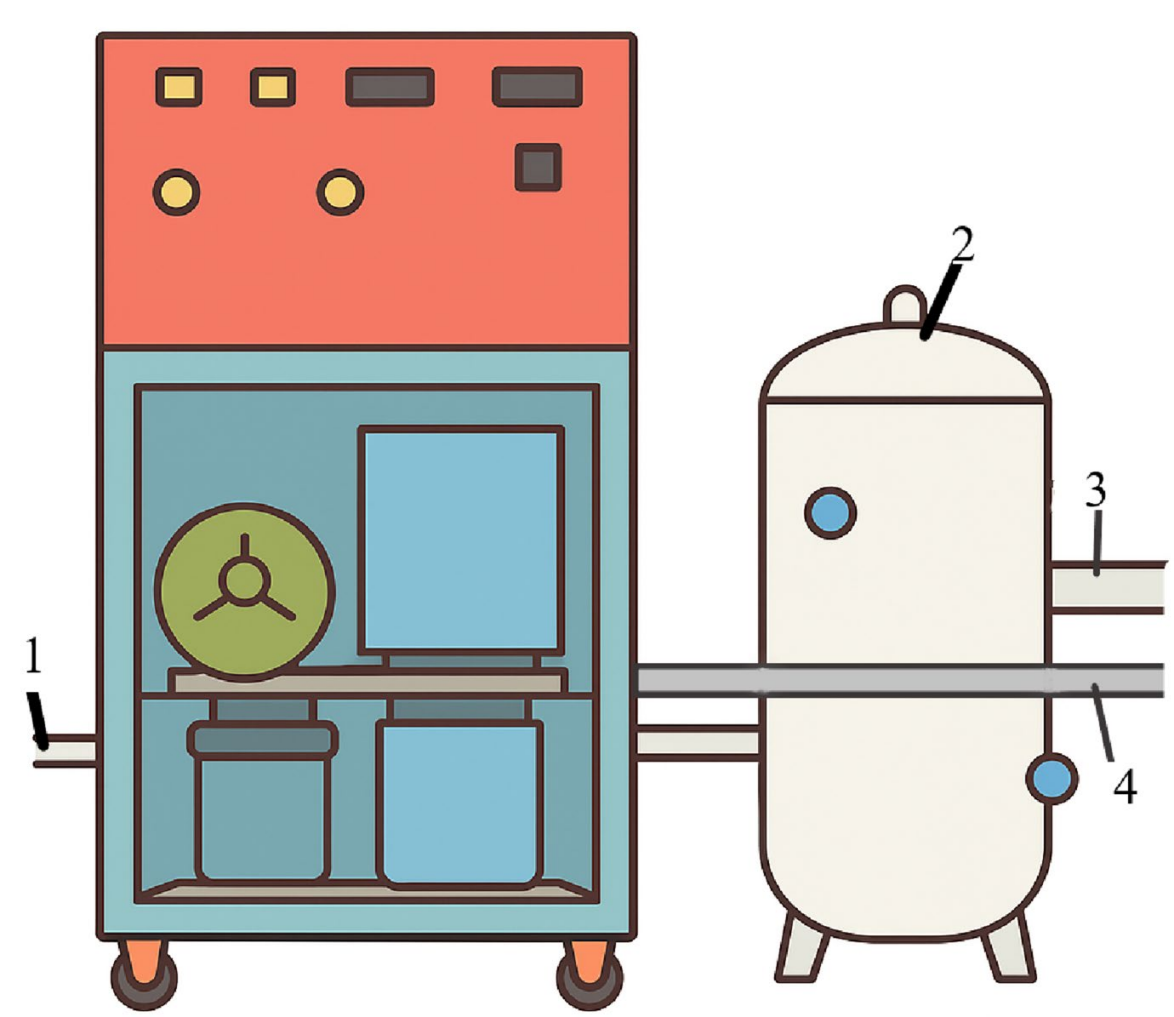
Experimental ion-ozone process line for the production of ion-ozonated water: 1––pipeline for connecting the compressor; 2––tank for ion-ozonating the water; 3––branch pipe for pouring out the ion-ozonated water; and 4––pipeline for creating excess pressure.

In this study, the main raw materials used were whole grain flour from durum wheat of the ‘Satti’ variety (Manufacturer: Kazakh Research Institute of Agriculture and Plant Growing, Almaty region, Almalybak village), carrot powder and ion-ozonated water. The ion-ozonated water was obtained using a laboratory ozone technical unit (Fig. 5) equipped with an ozone generator and an air ioniser. Ozonation was carried out at a C_io_ of 1.5–2.5 × 10^−6^ mg/cm^2^. This range of values corresponds to sanitary and technological recommendations for the food industry, ensuring controlled microbial contamination. It also improves the rheological properties of the dough^84,85^.

The ozone was quantitatively determined using the iodometric method in accordance with the GOST 18301-72 ‘Drinking water. Methods for determination of the residual ozone content standard^51^, which is based on the oxidation of potassium iodide with ozone, followed by titration of the released iodine with a sodium thiosulfate solution. The ions were determined by the titrimetric method in accordance with GOST R 58797–2020 ‘Water. Method for measuring the mass concentration of water-soluble ions’^52^, which provides for direct titration using standardised solutions.

The carrot powder was produced under laboratory conditions from selected Nantes carrots (Manufacturer: Peasant farm ‘Sharua’, Almaty region, Shamalgan village). The peeled and chopped carrots were dried at 60 °C for 10–12 h in a C-105 drying cabinet, then ground to a finely dispersed state (particle size ≤ 250 μm). This form promoted a uniform distribution of the additive in the dough and a more stable product quality than carrot juice^53–55^. The main characteristics of the carrot powder were a moisture content of 4.8%, a β-carotene content of 12.3 mg/100 g and an ash content of 5.6%.

Pasta samples were produced under laboratory conditions from the whole grain wheat flour with the added carrot powder and ion-ozonated water. The process of making the pasta consisted of preparing the raw materials, kneading the dough, pressing the dough, cutting the raw product, drying it, cooling it and packaging the finished product.

To prepare the pasta dough, a laboratory pasta press (AML-1, Russia) was used, which comprised a dough mixing chamber, a chamber for pressing the pasta, a gearbox and an electric motor. The kneader allowed dough to be kneaded from flour weighing from 300 to 1,500 g and was equipped with three kneading blades on a horizontal shaft that turned at 90 rpm. The chamber for pressing the pasta was located under the dough mixing chamber and was connected to it by means of a square opening equipped with a valve. The chamber for pressing the pasta had a pressure auger and a bronze matrix with a fluoroplastic insert. For the dough, 600 g of flour was placed in the dough mixer, the unit was turned on and the required amount of water was gradually added so that it was evenly distributed over the entire surface of the flour. When the dough was ready, the valve was opened and, using the kneading blades, the dough was moved into the pressing chamber, where it was fed by a screw onto the matrix and pressed into pasta. The pressed strands of pasta were placed on the table, cut into pieces of the required length and placed in cassettes for drying. The pasta was dried in a 105 °C drying cabinet. The recipe and parameters for the technological process of preparing the pasta samples under laboratory conditions are given in Table 9.

**Table 9:** Recipe and parameters for making the pasta samples.

Raw material and process indicators	Quantity
Wholemeal flour (kg)	100
Carrot powder (kg)	1–3
Ion-ozonated water (mL)	By calculation
t_w_ (°C), no more than	50–60
t_d_ (°C), ± 2	50–60

To obtain the ion-ozonated water, an experimental ion-ozonated technological line (Fig. 5) for the production of ion-ozonated water was used.

**Fig. 6:**
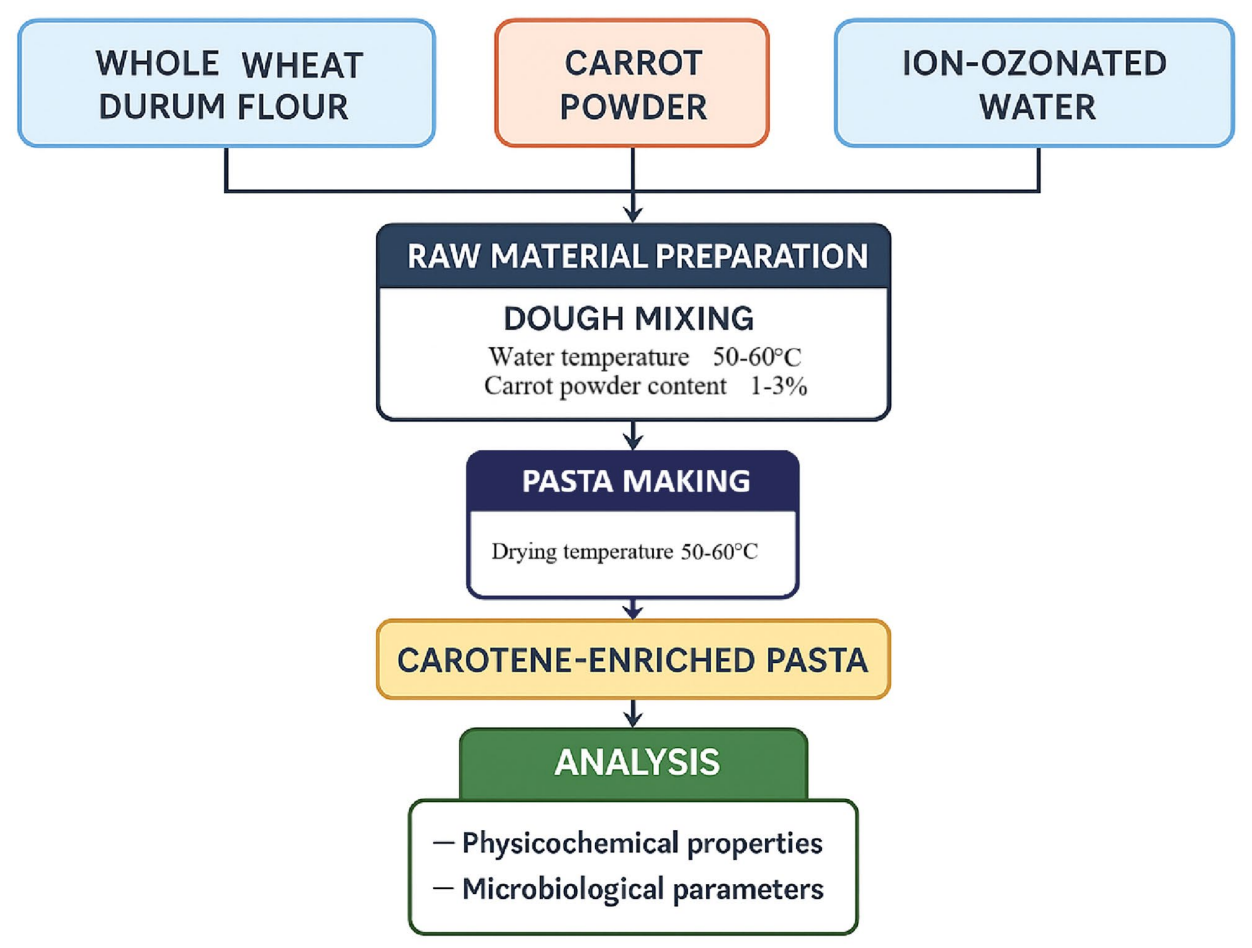
Scheme showing the technological process and experimental plan of the study.

The algorithm of the experimental study is shown in Fig. 6.

All tests were carried out in triplicate. The analyses were performed to state standards: humidity according to GOST 31964-2012^56^, drying cabinet SESh-3 M, 130 °C, 40 min; acidity by titration with sodium hydroxide according to GOST 31964-2012^56^; ash content according to GOST 31964-2012^56^, using hydrochloric acid treatment and a muffle furnace; shape retention by visual assessment of cooked 50 g samples according to GOST 31964-2012^56^; loss of DM by accelerated drying according to GOST 31964-2012^56^; weight and volume increase coefficients by calculation using the standard formula; duration of cooking until done based on the disappearance of the floury layer; protein content according to GOST 10846-91^57^ using the Kjeldahl method; fat content according to GOST 29033-91^58^ using extraction with preliminary hydrolysis; starch content according to GOST 10845-98^59^ using polarimetry; fibre content according to GOST 31675-2012^60^ using acid–base treatment; minerals (Fe, Ca, Mg, Zn, K) according to GOST 32343-2013^61^ using atomic absorption spectrometry; and vitamin contents (A, E, C, β-carotene) using high-performance liquid chromatography (Agilent ChemStation, Sigma-Aldrich calibration) with photometric and fluorimetric detection depending on the compound being analysed, according to GOST R 54635-2011^62^, GOST 34151-2017^63^, GOST EN 12822-2014^64^ and GOST EN 12823-2-2014^65^. This method offered the following detection limits: vitamin A (retinol)—0.01 mg/100 g; vitamin E (tocopherol)—0.02 mg/100 g; vitamin C (ascorbic acid)—0.05 mg/100 g; and β-carotene—0.02 mg/100 g. The extraction of vitamins from the pasta matrix was assessed using the standard addition method. The average recovery values were: vitamin A—92–95%; vitamin E—90–94%; vitamin C—87–91%; and β carotene—88–93%.

The methods were validated for the following parameters: linearity, where the correlation coefficients of the calibration curves were R^2^ > 0.995 for all the vitamins studied; repeatability, the relative standard deviation not exceeding 5%; accuracy, the relative deviation being no more than ± 7%; specificity, confirmed by the absence of cross-peaks in the analysis of the samples and standards. The calibration standards were obtained from Sigma-Aldrich. Data processing was performed using Agilent ChemStation software.

Data processing and all necessary calculations were carried out using the PLAN sequential regression analysis program developed at the Odessa National Academy of Food Technologies^34^. This program calculated the regression coefficients for each quality indicator, checked the significance of the regression coefficients and, after removing all insignificant coefficients, determined the necessary statistical characteristics of the obtained regression equations, including checking their adequacy with regard to the experimental data. The calculations of the regression coefficients were performed using matrices in natural dimensions and, accordingly, the equations themselves were also obtained in natural dimensions.

The general form of the equations for the obtained regression equations is as follows (for four factors):

4 $$\begin{array}{ccccc}{\rm{y }} & = {\rm{ b}}0{\rm{ }} + {\rm{ b1C}} + {\rm{ b2}} + {\rm{ b3w}} + {\rm{ b4}}\tau \\& \quad + {\rm{ b12}}{\rm{vo}} + {\rm{ b13Cvow}} + {\rm{ b14Cvo}}\tau \\& \quad + {\rm{ b23Pvow}} + {\rm{b24Pvo}}\tau + {\rm{b34wvo}}\tau \end{array}$$

where y represents the quality indicators of the pasta, C is the ratio of ion concentration (units/cm^3^) to ozone concentration (g/cm^3^) (units/g), P is the excess pressure (cavitation) (atm), w is the humidity before processing the pasta (%) and τ is the processing time (min).

The following quality indicators were determined for the pasta: physicochemical properties (y_1_–y_7_), rheological properties (y_8_–y_10_) and chemical composition (y_11_–y_25_). For the numerical evaluation of the coefficients in the regression equations in the PLAN program, the least squares method, implemented in matrix form, was used.

Because the experiments were executed once, to evaluate the dispersion of the reproducibility of the results in the centre of each studied sample, duplicate (parallel) experiments were performed. Based on the results of these, we determined the dispersion of reproducibility and, accordingly, the standard errors (s_y_) of the 16 experiments.

The significance of the coefficients (b_i_) was checked using the confidence interval, which was calculated using the formula:

5 $${\varepsilon _{{b_i}}} = {t_{cr}}\sqrt {S_{{b_i}}^2}$$

where${S_{{b_i}}^2}$ is the dispersion of the i-th regression coefficient. If the condition $\left| {{b_i}} \right| \ge {\varepsilon _{{b_i}}}$ was met, the coefficient was considered statistically significant for the adopted significance level (0.05).

Because the regression coefficients were calculated using matrices in natural dimensions, after removing the most insignificant coefficient, the remaining significant coefficients and their confidence intervals were recalculated. In accordance with the principle of sequential regression analysis, this procedure was performed until there was not a single insignificant coefficient left in the regression equation. The resulting regression equation was estimated by the relative errors between the empirical values and those calculated from it (%):

6 $$\delta = \frac{{\left| {\bar y - \hat y} \right|}}{{\bar y}}100,\%$$

However, more-accurate conclusions about the suitability of the obtained equation for practical use were made after its statistical analysis based on the dispersion estimates. For this purpose, the obtained equation, from which all insignificant coefficients were excluded, was checked for adequacy against the experimental data. Adequacy testing was performed using the Fisher criterion, which is the ratio of the larger dispersion to the smaller dispersion:

7 $$F = \frac{{S_{{inad}}^{2} }}{{S\frac{2}{y}}}\left( {at\,S_{{inad}}^{2} > S\frac{2}{y}} \right)\,{\mathrm{or}}\,F = \frac{{S\frac{2}{y}}}{{S_{{inad}}^{2} }}\left( {at\,S\frac{2}{y} > S_{{inad}}^{2} } \right)$$

where $\:{S}_{\mathrm{inad}}^{2}$ is the variance of inadequacy, which characterises the spread of the values calculated using the equation $\hat{y}_{u}$ and the results of the experiments (measurements) $\bar{y}_{u}$. $S_{{\overline{y} }}^{2}$ is the variance of the experimental error, which characterises the spread of the values of y_u_ in the parallel experiments and the average value of the experiments $\overline{y} _{u}$.

The calculated value of the Fisher criterion obtained using one of the above expressions was compared with the tabulated (critical) value, F_cr_. In the case where F < F_cr_, the resulting equation adequately described the experimental data within the accepted reliability of 95%.

All regression coefficients for the pasta samples, as well as their statistical characteristics, were obtained from PLAN^64^. The following notations were used in the PLAN program listings: b––regression coefficients; e––confidence intervals for the corresponding coefficients; x_1_, х_2_, х_3_, х_4_, …––designations of the first, second, third and fourth, etc., factors; Y_av_––experimental values of quality indicators Y; Y_c_––values of the quality indicator Y, calculated according to the obtained regression equation, from which all insignificant coefficients have been removed; styu––relative deviation (in %) of the calculated Y_c_ and experimental Y_av_ values of the quality indicator Y; t_cr_––critical (tabular) value of the student’s criterion (for a significance level of 0.05); s2y, s2ag––weighted average variance of the mean of the experimental results, s2y, and the variance of inadequacy, s2ad, respectively; sy, sag––the same, but standard, deviations; Ns2y, Ns2ag––number of degrees of freedom, as above; and F_c_, F_cr_––calculated and critical (tabular) values of the Fisher criterion. The adequacy of the equations to the experimental data was checked for a significance level of 0.05.

The microbiological safety of the pasta samples was evaluated in accordance with the requirements of GOST 10444.15-94 “Food products. Methods for determining the quantity of mesophilic aerobic and facultative anaerobic microorganisms”^66^, GOST 10444.12–2013 “Food products. Methods for detection of yeast and mould”^61^, and GOST 31747-2012 “Methods for detecting and determining the number of coliform bacteria”^67^.

The results showed no detectable microbial growth in the pasta samples after production and drying. This outcome confirms the hygienic efficiency of ion-ozonated water, which effectively destroys vegetative forms of microorganisms and spores on the surface of raw materials and equipment. Therefore, ion-ozonated water contributes significantly to the microbiological safety of the final pasta product and supports its classification as a clean-label food technology.

### Statistical analysis

The experimental data were processed using variation and regression statistics. The main analysis was carried out using Statistica version 10.0 software (StatSoft, USA) and MS Excel version 10 Pro (2010) and the Solver tool. Additionally, to clarify the parameters of the regression models and build confidence intervals, we used modern analytical tools on the Python platform (statsmodels library). Both absolute and relative statistical indicators and tabular and graphical methods for presenting the results were used in the analysis. The values were calculated based on the arithmetic means obtained under the conditions of experiments performed in triplicate. To assess the organoleptic characteristics, questionnaires were used with a tasting group. The constructed regression models made it possible to quantitatively describe the influence of technological factors on the output characteristics of the pasta products. Standard errors, p values and 95% confidence intervals were calculated for the regression coefficients, which confirmed their statistical significance and the stability of the models. The p values for all key factors were below 0.05, indicating a high level of reliability of the identified dependencies. Given the presence of significant pairwise interaction coefficients reflecting the nonlinear nature of the dependence of quality indicators on factors, optimisation of the process modes was carried out using nonlinear programming methods. In particular, the Newton method implemented in the Solver module of MS Excel was used, which made it possible to effectively determine the conditions that provide the best values for the target parameters, such as protein content and minimal DM loss.

## Conclusion

The aim of this study was to optimise the technological modes of durum wheat pasta production using carrot powder and ion-ozonated water. A multifactorial experimental design and regression analysis were used, aimed at assessing the impact of key technological parameters on the quality of the finished products––particularly protein content and DM loss during cooking.

We found that minimal DM loss (5.86%) occurred with pasta having a C_cp._ of 3.0%, dried at 50 °C, the maximum protein content (14.25%) was achieved with a C_cp._ of 1.0% and drying at 50 °C, and the C_io_ and the t_w_ did not statistically significantly effect these parameters. The data confirm the possibility for the targeted control of the quality indicators of pasta by regulating the composition of the plant additive and the t_d_.

The novelty of this work lies in the clarification of the influence of factors that have not previously received sufficient experimental justification in the context of the use of ion-ozonated water and carrot powder in pasta production. The results are of great practical importance and can be used in the development of technological regulations and recipes for functional pasta products. The proposed approaches ensure an increase in nutritional value without deteriorating the technological properties of the product. In further studies, we suggest assessing the environmental and economic efficiency of the introduction of ion-ozonated water on an industrial scale and studying consumer preferences and their organoleptic perception of the fortified products using focus groups. These areas can become the basis for the further development of sustainable and functional products that meet the modern requirements of the food industry and consumers.

## Industrial applicability and raw material variability

The developed technology is suitable for industrial implementation without the need for equipment modification. Any adjustments relate only to the drying regimes and dough formulation, which can easily be managed on standard pasta production lines. Incorporating carrot powder at concentrations of 1.0–3.0% does not impair the technological properties of the dough and, in fact, enhances the nutritional value of the final product. The pasta retains its shape and complies with regulatory standards for DM loss during cooking.

Given that the quality of the raw materials (especially the carrot powder and whole wheat flour) may vary depending on harvest conditions, it is recommended to apply incoming raw material control and adjust the processing parameters accordingly. This approach would ensure consistent product quality regardless of seasonal fluctuations.

## Supplementary Information

Below is the link to the electronic supplementary material.

Supplementary Material 1

## Data Availability

For additional information, please contact the corresponding author, Dr. Meruet Baiysbayeva (e-mail: meruyet-80@rambler.ru). Data will be made available on request. An open-access repository containing the original experimental data and the scripts used in the PLAN software for multifactor analysis is available at the following link: https://docs.google.com/document/d/1hqSehhrshk1Q3qxgHq8zdTXV9TvarkqG/edit? usp=sharing&ouid=106391404304730154262&rtpof=true&sd=true.
