# Supplementary material for: Optimization of technological parameters for durum wheat pasta production with carrot powder and ion-ozonated water
Source: Sci Rep. 2025 Dec 24;15:44511. doi: 10.1038/s41598-025-28027-0 (PMC12738575; doi:10.1038/s41598-025-28027-0)
Supplement: Supplementary file 1 — Supplementary Material 1 [file 41598_2025_28027_MOESM1_ESM.docx]

APPENDIX A

CALCULATION AND STATISTICAL EVALUATION OF REGRESSION EQUATIONS DESCRIBING QUALITY INDICATORS OF PASTA PRODUCTS MADE FROM SATTI WHEAT FLOUR

CALCULATION OF REGRESSION COEFFICIENTS BY THE LEAST SQUARES METHOD FOR A LINEAR PLAN WITH INTERFACTOR INTERACTIONS

Number of experiments N= 16, coefficients KK=11, factors KF= 4

Х1 - Cо*10^-6, mg/unit.; Х2 - Tw, °C С; Х3 - Ccp, %; Х4 - Td, °C

У1 - Humidity, %

Number of replicates of experiments m = 1 m0= 0

Regression coefficients (b) and their confidence errors (e):

b0 =16.60000 b1 =-0.65000 b2 =-0.02000 b3 = 0.12500

b4 =-0.04500 b12= 0.01000 b13= 0.05000 b14= 0.00000

b23=-0.00500 b24=-0.00000 b34=-0.00000

e0 =34.97849 e1 = 7.81195 e2 = 0.59232 e3 = 3.99973

e4 = 0.59232 e12= 0.09941 e13= 0.49706 e14= 0.09941

e23= 0.04971 e24= 0.00994 e34= 0.04971

Significant regression coefficients:

b0 =12.72500

e0 = 0.24853

------------------------------------------------------------------

N X1 X2 X3 X4 Yavg Yr styp

------------------------------------------------------------------

1 2.500 60.000 3.000 60.000 12.60 12.73 0.99

2 1.500 60.000 3.000 60.000 12.20 12.73 4.30

3 2.500 50.000 3.000 60.000 12.40 12.73 2.62

4 1.500 50.000 3.000 60.000 12.60 12.73 0.99

5 2.500 60.000 1.000 60.000 12.40 12.73 2.62

6 1.500 60.000 1.000 60.000 12.60 12.73 0.99

7 2.500 50.000 1.000 60.000 12.60 12.73 0.99

8 1.500 50.000 1.000 60.000 12.60 12.73 0.99

9 2.500 60.000 3.000 50.000 12.80 12.73 0.59

10 1.500 60.000 3.000 50.000 12.80 12.73 0.59

11 2.500 50.000 3.000 50.000 13.00 12.73 2.12

12 1.500 50.000 3.000 50.000 13.00 12.73 2.12

13 2.500 60.000 1.000 50.000 13.00 12.73 2.12

14 1.500 60.000 1.000 50.000 13.00 12.73 2.12

15 2.500 50.000 1.000 50.000 13.00 12.73 2.12

16 1.500 50.000 1.000 50.000 13.00 12.73 2.12

------------------------------------------------------------------

min 1.500 50.000 1.000 50.000 12.73

max 1.500 50.000 1.000 50.000 12.73

Statistical indicators:

Student’s criterion tcr= 4.304

variance of error of experience and inadequacy s2y= 0.0534 s2ag= 0.0687

standard deviation sy = 0.2310 sag = 0.2620

degrees of freedom Ns2y= 2 Ns2ag= 15

Fisher’s criterion Fr= 1.29 Fcr= 19.43

CALCULATION OF REGRESSION COEFFICIENTS BY THE LEAST SQUARES METHOD FOR A LINEAR PLAN WITH INTERFACTOR INTERACTIONS

Number of experiments N= 16, coefficients KK=11, factors KF= 4

Х1 - Cо*10^-6, mg/unit.; Х2 - Tw, °C С; Х3 - Ccp, %; Х4 - Td, °C

У2 - Acidity, degrees

Number of replicates of experiments m = 1 m0= 0

Regression coefficients (b) and their confidence errors (e):

b0 = 9.68000 b1 =-1.98500 b2 =-0.08050 b3 =-0.41250

b4 =-0.06300 b12= 0.01800 b13= 0.08500 b14= 0.01300

b23= 0.00100 b24= 0.00080 b34= 0.00350

e0 =27.25596 e1 = 6.08723 e2 = 0.46155 e3 = 3.11667

e4 = 0.46155 e12= 0.07746 e13= 0.38732 e14= 0.07746

e23= 0.03873 e24= 0.00775 e34= 0.03873

Significant regression coefficients:

b0 = 3.65750

e0 = 0.19366

------------------------------------------------------------------

N X1 X2 X3 X4 Yavg Yr styp

------------------------------------------------------------------

1 2.500 60.000 3.000 60.000 4.00 3.66 8.56

2 1.500 60.000 3.000 60.000 3.80 3.66 3.75

3 2.500 50.000 3.000 60.000 3.60 3.66 1.60

4 1.500 50.000 3.000 60.000 3.60 3.66 1.60

5 2.500 60.000 1.000 60.000 3.62 3.66 1.04

6 1.500 60.000 1.000 60.000 3.60 3.66 1.60

7 2.500 50.000 1.000 60.000 3.60 3.66 1.60

8 1.500 50.000 1.000 60.000 4.00 3.66 8.56

9 2.500 60.000 3.000 50.000 3.40 3.66 7.57

10 1.500 60.000 3.000 50.000 3.50 3.66 4.50

11 2.500 50.000 3.000 50.000 3.60 3.66 1.60

12 1.500 50.000 3.000 50.000 3.80 3.66 3.75

13 2.500 60.000 1.000 50.000 3.60 3.66 1.60

14 1.500 60.000 1.000 50.000 3.80 3.66 3.75

15 2.500 50.000 1.000 50.000 3.40 3.66 7.57

16 1.500 50.000 1.000 50.000 3.60 3.66 1.60

------------------------------------------------------------------

min 1.500 50.000 1.000 50.000 3.66

max 1.500 50.000 1.000 50.000 3.66

Statistical indicators:

Student’s criterion tcr= 4.304

variance of error of experience and inadequacy s2y= 0.0324 s2ag= 0.0318

standard deviation sy = 0.1800 sag = 0.1784

degrees of freedom Ns2y= 2 Ns2ag= 15

Fisher’s criterion Fr= 1.02 Fcr= 3.68

CALCULATION OF REGRESSION COEFFICIENTS BY THE LEAST SQUARES METHOD FOR A LINEAR PLAN WITH INTERFACTOR INTERACTIONS

Number of experiments N= 16, coefficients KK=11, factors KF= 4

Х1 - Cо*10^-6, mg/unit.; Х2 - Tw, °C С; Х3 - Ccp, %; Х4 - Td, °C

У3 - Shape retention, %

Number of replicates of experiments m = 1 m0= 0

Regression coefficients (b) and their confidence errors (e):

b0 =121.00000 b1 =-7.75000 b2 =-0.50000 b3 = 8.12500

b4 =-0.17500 b12= 0.05000 b13= 0.50000 b14= 0.10000

b23= 0.02500 b24= 0.00500 b34=-0.20000

e0 =439.12388 e1 =98.07204 e2 = 7.43603 e3 =50.21305

e4 = 7.43603 e12= 1.24803 e13= 6.24017 e14= 1.24803

e23= 0.62402 e24= 0.12480 e34= 0.62402

Significant regression coefficients:

b0 =99.00000

e0 = 3.12009

------------------------------------------------------------------

N X1 X2 X3 X4 Yavg Yr styp

------------------------------------------------------------------

1 2.500 60.000 3.000 60.000 98.00 99.00 1.02

2 1.500 60.000 3.000 60.000 95.00 99.00 4.21

3 2.500 50.000 3.000 60.000 100.00 99.00 1.00

4 1.500 50.000 3.000 60.000 95.00 99.00 4.21

5 2.500 60.000 1.000 60.000 100.00 99.00 1.00

6 1.500 60.000 1.000 60.000 100.00 99.00 1.00

7 2.500 50.000 1.000 60.000 100.00 99.00 1.00

8 1.500 50.000 1.000 60.000 100.00 99.00 1.00

9 2.500 60.000 3.000 50.000 100.00 99.00 1.00

10 1.500 60.000 3.000 50.000 100.00 99.00 1.00

11 2.500 50.000 3.000 50.000 100.00 99.00 1.00

12 1.500 50.000 3.000 50.000 100.00 99.00 1.00

13 2.500 60.000 1.000 50.000 100.00 99.00 1.00

14 1.500 60.000 1.000 50.000 96.00 99.00 3.13

15 2.500 50.000 1.000 50.000 100.00 99.00 1.00

16 1.500 50.000 1.000 50.000 100.00 99.00 1.00

------------------------------------------------------------------

min 1.500 50.000 1.000 50.000 99.00

max 1.500 50.000 1.000 50.000 99.00

Statistical indicators:

Student’s criterion tcr= 4.304

variance of error of experience and inadequacy s2y= 8.4100 s2ag= 3.6000

standard deviation sy = 2.9000 sag = 1.8974

degrees of freedom Ns2y= 2 Ns2ag= 15

Fisher’s criterion Fr= 2.34 Fcr= 3.68

CALCULATION OF REGRESSION COEFFICIENTS BY THE LEAST SQUARES METHOD FOR A LINEAR PLAN WITH INTERFACTOR INTERACTIONS

Number of experiments N= 16, coefficients KK=11, factors KF= 4

Х1 - Cо*10^-6, mg/unit.; Х2 - Tw, °C С; Х3 - Ccp, %; Х4 - Td, °C

У4 - Mass increase coefficient (Cm)

Number of replicates of experiments m = 1 m0= 0

Regression coefficients (b) and their confidence errors (e):

b0 = 0.61000 b1 = 0.47500 b2 = 0.03625 b3 =-0.49500

b4 = 0.00700 b12=-0.00550 b13=-0.07500 b14=-0.00200

b23=-0.00225 b24=-0.00035 b34= 0.01350

e0 = 7.87395 e1 = 1.75853 e2 = 0.13334 e3 = 0.90037

e4 = 0.13334 e12= 0.02238 e13= 0.11189 e14= 0.02238

e23= 0.01119 e24= 0.00224 e34= 0.01119

Significant regression coefficients:

b0 = 2.05500 b3 =-0.41125 b34= 0.00700

e0 = 0.12510 e3 = 0.28085 e34= 0.00500

------------------------------------------------------------------

N X1 X2 X3 X4 Yavg Yr styp

------------------------------------------------------------------

1 2.500 60.000 3.000 60.000 1.97 2.08 5.65

2 1.500 60.000 3.000 60.000 2.18 2.08 4.53

3 2.500 50.000 3.000 60.000 2.01 2.08 3.54

4 1.500 50.000 3.000 60.000 2.23 2.08 6.67

5 2.500 60.000 1.000 60.000 2.04 2.06 1.16

6 1.500 60.000 1.000 60.000 2.03 2.06 1.66

7 2.500 50.000 1.000 60.000 2.01 2.06 2.67

8 1.500 50.000 1.000 60.000 1.98 2.06 4.23

9 2.500 60.000 3.000 50.000 1.82 1.87 2.82

10 1.500 60.000 3.000 50.000 1.92 1.87 2.54

11 2.500 50.000 3.000 50.000 1.78 1.87 5.13

12 1.500 50.000 3.000 50.000 1.90 1.87 1.51

13 2.500 60.000 1.000 50.000 1.98 1.99 0.69

14 1.500 60.000 1.000 50.000 2.14 1.99 6.83

15 2.500 50.000 1.000 50.000 2.06 1.99 3.22

16 1.500 50.000 1.000 50.000 1.99 1.99 0.19

------------------------------------------------------------------

min 1.500 50.000 3.000 50.000 1.87

max 1.500 50.000 3.000 60.000 2.08

Statistical indicators:

Student’s criterion tcr= 4.304

variance of error of experience and inadequacy s2y= 0.0027 s2ag= 0.0078

standard deviation sy = 0.0520 sag = 0.0882

degrees of freedom Ns2y= 2 Ns2ag= 13

Fisher’s criterion Fr= 2.88 Fcr= 19.42

CALCULATION OF REGRESSION COEFFICIENTS BY THE LEAST SQUARES METHOD FOR A LINEAR PLAN WITH INTERFACTOR INTERACTIONS

Number of experiments N= 16, coefficients KK=11, factors KF= 4

Х1 - Cо*10^-6, mg/unit.; Х2 - Tw, °C С; Х3 - Ccp, %; Х4 - Td, °C

У5 - Volume increase coefficient (Cv)

Number of replicates of experiments m = 1 m0= 0

Regression coefficients (b) and their confidence errors (e):

b0 =-0.12000 b1 = 0.37500 b2 = 0.02800 b3 =-0.38875

b4 = 0.01150 b12=-0.00400 b13=-0.01750 b14=-0.00300

b23=-0.00075 b24=-0.00030 b34= 0.00825

e0 =18.17064 e1 = 4.05815 e2 = 0.30770 e3 = 2.07778

e4 = 0.30770 e12= 0.05164 e13= 0.25821 e14= 0.05164

e23= 0.02582 e24= 0.00516 e34= 0.02582

Significant regression coefficients:

b0 = 1.10250

e0 = 0.12911

------------------------------------------------------------------

N X1 X2 X3 X4 Yavg Yr styp

------------------------------------------------------------------

1 2.500 60.000 3.000 60.000 1.10 1.10 0.23

2 1.500 60.000 3.000 60.000 1.23 1.10 10.37

3 2.500 50.000 3.000 60.000 1.10 1.10 0.23

4 1.500 50.000 3.000 60.000 1.21 1.10 8.88

5 2.500 60.000 1.000 60.000 1.10 1.10 0.23

6 1.500 60.000 1.000 60.000 1.10 1.10 0.23

7 2.500 50.000 1.000 60.000 1.10 1.10 0.23

8 1.500 50.000 1.000 60.000 1.10 1.10 0.23

9 2.500 60.000 3.000 50.000 1.02 1.10 8.09

10 1.500 60.000 3.000 50.000 1.04 1.10 6.01

11 2.500 50.000 3.000 50.000 1.02 1.10 8.09

12 1.500 50.000 3.000 50.000 1.01 1.10 9.16

13 2.500 60.000 1.000 50.000 1.10 1.10 0.23

14 1.500 60.000 1.000 50.000 1.21 1.10 8.88

15 2.500 50.000 1.000 50.000 1.10 1.10 0.23

16 1.500 50.000 1.000 50.000 1.10 1.10 0.23

------------------------------------------------------------------

min 1.500 50.000 1.000 50.000 1.10

max 1.500 50.000 1.000 50.000 1.10

Statistical indicators:

Student’s criterion tcr= 4.304

variance of error of experience and inadequacy s2y= 0.0144 s2ag= 0.0044

standard deviation sy = 0.1200 sag = 0.0661

degrees of freedom Ns2y= 2 Ns2ag= 15

Fisher’s criterion Fr= 3.30 Fcr= 3.68

CALCULATION OF REGRESSION COEFFICIENTS BY THE LEAST SQUARES METHOD FOR A LINEAR PLAN WITH INTERFACTOR INTERACTIONS

Number of experiments N= 16, coefficients KK=11, factors KF= 4

Х1 - Cо*10^-6, mg/unit.; Х2 - Tw, °C С; Х3 - Ccp, %; Х4 - Td, °C

У6 - Amount of dry matter transferred to cooking water, %

Number of replicates of experiments m = 1 m0= 0

Regression coefficients (b) and their confidence errors (e):

b0 = 1.03375 b1 = 1.10125 b2 = 0.11025 b3 =-0.88188

b4 = 0.04750 b12=-0.01225 b13=-0.08125 b14=-0.00775

b23=-0.00538 b24=-0.00122 b34= 0.02437

e0 =15.44505 e1 = 3.44943 e2 = 0.26154 e3 = 1.76611

e4 = 0.26154 e12= 0.04390 e13= 0.21948 e14= 0.04390

e23= 0.02195 e24= 0.00439 e34= 0.02195

Significant regression coefficients:

b0 = 6.00687 b3 =-0.56175 b34= 0.01022

e0 = 0.24539 e3 = 0.55090 e34= 0.00982

------------------------------------------------------------------

N X1 X2 X3 X4 Yavg Yr styp

------------------------------------------------------------------

1 2.500 60.000 3.000 60.000 6.00 6.16 2.70

2 1.500 60.000 3.000 60.000 6.40 6.16 3.72

3 2.500 50.000 3.000 60.000 5.98 6.16 3.05

4 1.500 50.000 3.000 60.000 6.41 6.16 3.87

5 2.500 60.000 1.000 60.000 5.98 6.06 1.31

6 1.500 60.000 1.000 60.000 5.95 6.06 1.83

7 2.500 50.000 1.000 60.000 5.94 6.06 2.00

8 1.500 50.000 1.000 60.000 5.94 6.06 2.00

9 2.500 60.000 3.000 50.000 5.81 5.86 0.78

10 1.500 60.000 3.000 50.000 5.87 5.86 0.25

11 2.500 50.000 3.000 50.000 5.76 5.86 1.66

12 1.500 50.000 3.000 50.000 5.84 5.86 0.26

13 2.500 60.000 1.000 50.000 5.95 5.96 0.11

14 1.500 60.000 1.000 50.000 6.41 5.96 7.08

15 2.500 50.000 1.000 50.000 6.00 5.96 0.73

16 1.500 50.000 1.000 50.000 5.89 5.96 1.13

------------------------------------------------------------------

min 1.500 50.000 3.000 50.000 5.86

max 1.500 50.000 3.000 60.000 6.16

Statistical indicators:

Student’s criterion tcr= 4.304

variance of error of experience and inadequacy s2y= 0.0104 s2ag= 0.0344

standard deviation sy = 0.1020 sag = 0.1855

degrees of freedom Ns2y= 2 Ns2ag= 13

Fisher’s criterion Fr= 3.31 Fcr= 19.42

CALCULATION OF REGRESSION COEFFICIENTS BY THE LEAST SQUARES METHOD FOR A LINEAR PLAN WITH INTERFACTOR INTERACTIONS

Number of experiments N= 16, coefficients KK=11, factors KF= 4

Х1 - Cо*10^-6, mg/unit.; Х2 - Tw, °C С; Х3 - Ccp, %; Х4 - Td, °C

У7 - Cooking time until doneness, min

Number of replicates of experiments m = 1 m0= 0

Regression coefficients (b) and their confidence errors (e):

b0 =30.75000 b1 =-2.50000 b2 =-0.42500 b3 = 3.62500

b4 =-0.15000 b12= 0.05000 b13= 0.25000 b14=-0.00000

b23=-0.00000 b24= 0.00500 b34=-0.07500

e0 =43.91239 e1 = 9.80720 e2 = 0.74360 e3 = 5.02131

e4 = 0.74360 e12= 0.12480 e13= 0.62402 e14= 0.12480

e23= 0.06240 e24= 0.01248 e34= 0.06240

Significant regression coefficients:

b0 =14.75000 b3 =-0.70000 b13= 0.35000

e0 = 0.69767 e3 = 0.63943 e13= 0.27907

------------------------------------------------------------------

N X1 X2 X3 X4 Yavg Yr styp

------------------------------------------------------------------

1 2.500 60.000 3.000 60.000 15.00 15.27 1.83

2 1.500 60.000 3.000 60.000 13.00 14.22 9.42

3 2.500 50.000 3.000 60.000 15.00 15.27 1.83

4 1.500 50.000 3.000 60.000 14.00 14.22 1.61

5 2.500 60.000 1.000 60.000 15.00 14.92 0.50

6 1.500 60.000 1.000 60.000 15.00 14.57 2.83

7 2.500 50.000 1.000 60.000 15.00 14.92 0.50

8 1.500 50.000 1.000 60.000 15.00 14.57 2.83

9 2.500 60.000 3.000 50.000 15.00 15.27 1.83

10 1.500 60.000 3.000 50.000 15.00 14.22 5.17

11 2.500 50.000 3.000 50.000 16.00 15.27 4.53

12 1.500 50.000 3.000 50.000 15.00 14.22 5.17

13 2.500 60.000 1.000 50.000 15.00 14.92 0.50

14 1.500 60.000 1.000 50.000 13.00 14.57 12.12

15 2.500 50.000 1.000 50.000 15.00 14.92 0.50

16 1.500 50.000 1.000 50.000 15.00 14.57 2.83

------------------------------------------------------------------

min 1.500 50.000 3.000 50.000 14.22

max 2.500 50.000 3.000 50.000 15.27

Statistical indicators:

Student’s criterion tcr= 4.304

variance of error of experience and inadequacy s2y= 0.0841 s2ag= 0.5038

standard deviation sy = 0.2900 sag = 0.7098

degrees of freedom Ns2y= 2 Ns2ag= 13

Fisher’s criterion Fr= 5.99 Fcr= 19.42

CALCULATION OF REGRESSION COEFFICIENTS BY THE LEAST SQUARES METHOD FOR A LINEAR PLAN WITH INTERFACTOR INTERACTIONS

Number of experiments N= 16, coefficients KK=11, factors KF= 4

Х1 - Cо*10^-6, mg/unit.; Х2 - Tw, °C С; Х3 - Ccp, %; Х4 - Td, °C

У8 - Total deformation H1, mm

Number of replicates of experiments m = 1 m0= 0

Regression coefficients (b) and their confidence errors (e):

b0 =42.88250 b1 =-0.89750 b2 =-0.23700 b3 =-11.66125

b4 =-0.77700 b12=-0.02350 b13= 0.03000 b14= 0.04350

b23= 0.00025 b24= 0.00500 b34= 0.21175

e0 =107.81248 e1 =24.07838 e2 = 1.82567 e3 =12.32817

e4 = 1.82567 e12= 0.30641 e13= 1.53207 e14= 0.30641

e23= 0.15321 e24= 0.03064 e34= 0.15321

Significant regression coefficients:

b0 =25.46750 b3 =-11.58750 b4 =-0.41500 b34= 0.21175

e0 =18.91967 e3 = 8.46114 e4 = 0.34258 e34= 0.15321

------------------------------------------------------------------

N X1 X2 X3 X4 Yavg Yr styp

------------------------------------------------------------------

1 2.500 60.000 3.000 60.000 4.59 3.92 14.60

2 1.500 60.000 3.000 60.000 3.70 3.92 5.95

3 2.500 50.000 3.000 60.000 4.05 3.92 3.21

4 1.500 50.000 3.000 60.000 3.34 3.92 17.37

5 2.500 60.000 1.000 60.000 1.62 1.68 4.01

6 1.500 60.000 1.000 60.000 1.63 1.68 3.37

7 2.500 50.000 1.000 60.000 1.91 1.68 11.78

8 1.500 50.000 1.000 60.000 1.58 1.68 6.65

9 2.500 60.000 3.000 50.000 1.48 1.72 16.05

10 1.500 60.000 3.000 50.000 1.34 1.72 28.17

11 2.500 50.000 3.000 50.000 1.74 1.72 1.29

12 1.500 50.000 3.000 50.000 2.31 1.72 25.65

13 2.500 60.000 1.000 50.000 3.47 3.72 7.13

14 1.500 60.000 1.000 50.000 3.91 3.72 4.92

15 2.500 50.000 1.000 50.000 4.27 3.72 12.94

16 1.500 50.000 1.000 50.000 3.22 3.72 15.45

------------------------------------------------------------------

min 1.500 50.000 1.000 60.000 1.68

max 1.500 50.000 3.000 60.000 3.92

Statistical indicators:

Student’s criterion tcr= 4.304

variance of error of experience and inadequacy s2y= 0.5069 s2ag= 0.1768

standard deviation sy = 0.7120 sag = 0.4204

degrees of freedom Ns2y= 2 Ns2ag= 12

Fisher’s criterion Fr= 2.87 Fcr= 3.89

CALCULATION OF REGRESSION COEFFICIENTS BY THE LEAST SQUARES METHOD FOR A LINEAR PLAN WITH INTERFACTOR INTERACTIONS

Number of experiments N= 16, coefficients KK=11, factors KF= 4

Х1 - Cо*10^-6, mg/unit.; Х2 - Tw, °C С; Х3 - Ccp, %; Х4 - Td, °C

У9 - Plastic deformation H2, mm

Number of replicates of experiments m = 1 m0= 0

Regression coefficients (b) and their confidence errors (e):

b0 =51.68125 b1 = 1.34125 b2 =-0.85475 b3 =-8.94563

b4 =-0.75500 b12=-0.00425 b13=-0.31875 b14= 0.01725

b23= 0.13587 b24= 0.01157 b34= 0.04462

e0 =51.48349 e1 =11.49810 e2 = 0.87181 e3 = 5.88705

e4 = 0.87181 e12= 0.14632 e13= 0.73161 e14= 0.14632

e23= 0.07316 e24= 0.01463 e34= 0.07316

Significant regression coefficients:

b0 =11.89875 b1 = 1.41875 b2 =-0.22663 b3 =-7.12875

b23= 0.13588

e0 = 9.15240 e1 = 0.73161 e2 = 0.16359 e3 = 4.04043

e23= 0.07316

------------------------------------------------------------------

N X1 X2 X3 X4 Yavg Yr styp

------------------------------------------------------------------

1 2.500 60.000 3.000 60.000 6.11 4.92 19.49

2 1.500 60.000 3.000 60.000 3.09 3.50 13.29

3 2.500 50.000 3.000 60.000 2.69 3.11 15.59

4 1.500 50.000 3.000 60.000 2.33 1.69 27.44

5 2.500 60.000 1.000 60.000 3.02 2.87 4.90

6 1.500 60.000 1.000 60.000 1.79 1.45 18.82

7 2.500 50.000 1.000 60.000 3.14 3.78 20.36

8 1.500 50.000 1.000 60.000 1.73 2.36 36.45

9 2.500 60.000 3.000 50.000 4.08 4.92 20.57

10 1.500 60.000 3.000 50.000 3.56 3.50 1.67

11 2.500 50.000 3.000 50.000 2.54 3.11 22.42

12 1.500 50.000 3.000 50.000 2.04 1.69 17.13

13 2.500 60.000 1.000 50.000 2.33 2.87 23.26

14 1.500 60.000 1.000 50.000 1.51 1.45 3.77

15 2.500 50.000 1.000 50.000 5.45 3.78 30.65

16 1.500 50.000 1.000 50.000 1.96 2.36 20.44

------------------------------------------------------------------

min 1.500 60.000 1.000 50.000 1.45

max 2.500 60.000 3.000 50.000 4.92

Statistical indicators:

Student’s criterion tcr= 4.304

variance of error of experience and inadequacy s2y= 0.1156 s2ag= 0.6832

standard deviation sy = 0.3400 sag = 0.8266

degrees of freedom Ns2y= 2 Ns2ag= 11

Fisher’s criterion Fr= 5.91 Fcr= 19.40

CALCULATION OF REGRESSION COEFFICIENTS BY THE LEAST SQUARES METHOD FOR A LINEAR PLAN WITH INTERFACTOR INTERACTIONS

Number of experiments N= 16, coefficients KK=11, factors KF= 4

Х1 - Cо*10^-6, mg/unit.; Х2 - Tw, °C С; Х3 - Ccp, %; Х4 - Td, °C

У10 - Elastic deformation H3, mm

Number of replicates of experiments m = 1 m0= 0

Regression coefficients (b) and their confidence errors (e):

b0 = 3.58500 b1 =-0.49500 b2 =-0.01150 b3 =-0.56125

b4 =-0.05125 b12= 0.00300 b13=-0.08250 b14= 0.01050

b23=-0.00175 b24= 0.00005 b34= 0.01550

e0 = 6.20830 e1 = 1.38654 e2 = 0.10513 e3 = 0.70991

e4 = 0.10513 e12= 0.01764 e13= 0.08822 e14= 0.01764

e23= 0.00882 e24= 0.00176 e34= 0.00882

Significant regression coefficients:

b0 = 2.29250 b3 =-0.82250 b4 =-0.02750 b34= 0.01550

e0 = 1.08948 e3 = 0.48723 e4 = 0.01973 e34= 0.00882

------------------------------------------------------------------

N X1 X2 X3 X4 Yavg Yr styp

------------------------------------------------------------------

1 2.500 60.000 3.000 60.000 1.15 0.96 16.09

2 1.500 60.000 3.000 60.000 0.84 0.96 14.88

3 2.500 50.000 3.000 60.000 0.86 0.96 12.21

4 1.500 50.000 3.000 60.000 1.01 0.96 4.46

5 2.500 60.000 1.000 60.000 0.71 0.75 5.63

6 1.500 60.000 1.000 60.000 0.61 0.75 22.95

7 2.500 50.000 1.000 60.000 0.98 0.75 23.47

8 1.500 50.000 1.000 60.000 0.70 0.75 7.14

9 2.500 60.000 3.000 50.000 0.72 0.78 7.64

10 1.500 60.000 3.000 50.000 0.61 0.78 27.05

11 2.500 50.000 3.000 50.000 0.75 0.78 3.33

12 1.500 50.000 3.000 50.000 1.02 0.78 24.02

13 2.500 60.000 1.000 50.000 0.85 0.87 2.35

14 1.500 60.000 1.000 50.000 0.98 0.87 11.22

15 2.500 50.000 1.000 50.000 1.03 0.87 15.53

16 1.500 50.000 1.000 50.000 0.62 0.87 40.32

------------------------------------------------------------------

min 1.500 50.000 1.000 60.000 0.75

max 1.500 50.000 3.000 60.000 0.96

Statistical indicators:

Student’s criterion tcr= 4.304

variance of error of experience and inadequacy s2y= 0.0017 s2ag= 0.0276

standard deviation sy = 0.0410 sag = 0.1661

degrees of freedom Ns2y= 2 Ns2ag= 12

Fisher’s criterion Fr= 16.41 Fcr= 19.41

CALCULATION OF REGRESSION COEFFICIENTS BY THE LEAST SQUARES METHOD FOR A LINEAR PLAN WITH INTERFACTOR INTERACTIONS

Number of experiments N= 16, coefficients KK=11, factors KF= 4

Х1 - Cо*10^-6, mg/unit.; Х2 - Tw, °C С; Х3 - Ccp, %; Х4 - Td, °C

У11- Protein, %

Number of replicates of experiments m = 1 m0= 0

Regression coefficients (b) and their confidence errors (e):

b0 =16.03250 b1 = 2.49750 b2 =-0.05400 b3 = 0.16625

b4 =-0.08200 b12=-0.02650 b13= 0.08250 b14=-0.02050

b23=-0.00975 b24= 0.00205 b34=-0.00375

e0 =12.26518 e1 = 2.73925 e2 = 0.20770 e3 = 1.40250

e4 = 0.20770 e12= 0.03486 e13= 0.17429 e14= 0.03486

e23= 0.01743 e24= 0.00349 e34= 0.01743

Significant regression coefficients:

b0 =15.55500 b3 =-0.41125 b4 =-0.01775

e0 = 0.97823 e3 = 0.08715 e4 = 0.01743

------------------------------------------------------------------

N X1 X2 X3 X4 Yavg Yr styp

------------------------------------------------------------------

1 2.500 60.000 3.000 60.000 13.20 13.26 0.43

2 1.500 60.000 3.000 60.000 13.24 13.26 0.12

3 2.500 50.000 3.000 60.000 13.22 13.26 0.27

4 1.500 50.000 3.000 60.000 13.29 13.26 0.25

5 2.500 60.000 1.000 60.000 14.07 14.08 0.06

6 1.500 60.000 1.000 60.000 14.09 14.08 0.08

7 2.500 50.000 1.000 60.000 14.13 14.08 0.36

8 1.500 50.000 1.000 60.000 14.10 14.08 0.15

9 2.500 60.000 3.000 50.000 13.19 13.43 1.85

10 1.500 60.000 3.000 50.000 13.28 13.43 1.16

11 2.500 50.000 3.000 50.000 14.09 13.43 4.66

12 1.500 50.000 3.000 50.000 13.25 13.43 1.39

13 2.500 60.000 1.000 50.000 14.18 14.26 0.54

14 1.500 60.000 1.000 50.000 14.25 14.26 0.04

15 2.500 50.000 1.000 50.000 14.28 14.26 0.17

16 1.500 50.000 1.000 50.000 14.24 14.26 0.11

------------------------------------------------------------------

min 1.500 50.000 3.000 60.000 13.26

max 1.500 50.000 1.000 50.000 14.26

Statistical indicators:

Student’s criterion tcr= 4.304

variance of error of experience and inadequacy s2y= 0.0066 s2ag= 0.0433

standard deviation sy = 0.0810 sag = 0.2082

degrees of freedom Ns2y= 2 Ns2ag= 13

Fisher’s criterion Fr= 6.60 Fcr= 19.42

CALCULATION OF REGRESSION COEFFICIENTS BY THE LEAST SQUARES METHOD FOR A LINEAR PLAN WITH INTERFACTOR INTERACTIONS

Number of experiments N= 16, coefficients KK=11, factors KF= 4

Х1 - Cо*10^-6, mg/unit.; Х2 - Tw, °C; Х3 - Ccp, %; Х4 - Td, °C

У12- Starch, %

Number of replicates of experiments m = 1 m0= 0

Regression coefficients (b) and their confidence errors (e):

b0 =83.30125 b1 =14.27875 b2 =-0.67375 b3 = 7.18312

b4 =-0.64275 b12=-0.14175 b13= 0.62625 b14=-0.12975

b23=-0.08288 b24= 0.01962 b34=-0.10787

e0 =187.76331 e1 =41.93425 e2 = 3.17954 e3 =21.47041

e4 = 3.17954 e12= 0.53364 e13= 2.66821 e14= 0.53364

e23= 0.26682 e24= 0.05336 e34= 0.26682

Significant regression coefficients:

b0 =68.95188 b3 =-2.05563

e0 = 2.98315 e3 = 1.33411

------------------------------------------------------------------

N X1 X2 X3 X4 Yavg Yr styp

------------------------------------------------------------------

1 2.500 60.000 3.000 60.000 62.12 62.79 1.07

2 1.500 60.000 3.000 60.000 62.56 62.79 0.36

3 2.500 50.000 3.000 60.000 61.97 62.79 1.32

4 1.500 50.000 3.000 60.000 61.56 62.79 1.99

5 2.500 60.000 1.000 60.000 67.42 66.90 0.78

6 1.500 60.000 1.000 60.000 67.58 66.90 1.01

7 2.500 50.000 1.000 60.000 66.98 66.90 0.13

8 1.500 50.000 1.000 60.000 66.99 66.90 0.14

9 2.500 60.000 3.000 50.000 61.95 62.79 1.35

10 1.500 60.000 3.000 50.000 61.98 62.79 1.30

11 2.500 50.000 3.000 50.000 67.55 62.79 7.05

12 1.500 50.000 3.000 50.000 62.59 62.79 0.31

13 2.500 60.000 1.000 50.000 66.78 66.90 0.17

14 1.500 60.000 1.000 50.000 66.59 66.90 0.46

15 2.500 50.000 1.000 50.000 66.35 66.90 0.82

16 1.500 50.000 1.000 50.000 66.48 66.90 0.63

------------------------------------------------------------------

min 1.500 50.000 3.000 50.000 62.79

max 1.500 50.000 1.000 50.000 66.90

Statistical indicators:

Student’s criterion tcr= 4.304

variance of error of experience and inadequacy s2y= 1.5376 s2ag= 2.0059

standard deviation sy = 1.2400 sag = 1.4163

degrees of freedom Ns2y= 2 Ns2ag= 14

Fisher’s criterion Fr= 1.30 Fcr= 19.42

CALCULATION OF REGRESSION COEFFICIENTS BY THE LEAST SQUARES METHOD FOR A LINEAR PLAN WITH INTERFACTOR INTERACTIONS

Number of experiments N= 16, coefficients KK=11, factors KF= 4

Х1 - Cо*10^-6, mg/unit.; Х2 - Tw, °C С; Х3 - Ccp, %; Х4 - Td, °C

Y13- Carbohydrate, %

Number of replicates of experiments m = 1 m0= 0

Regression coefficients (b) and their confidence errors (e):

b0 =80.22125 b1 =16.72625 b2 =-0.47200 b3 = 4.30063

b4 =-0.44825 b12=-0.15575 b13= 0.82625 b14=-0.16425

b23=-0.07913 b24= 0.01582 b34=-0.07488

e0 =231.67570 e1 =51.74146 e2 = 3.92314 e3 =26.49171

e4 = 3.92314 e12= 0.65845 e13= 3.29223 e14= 0.65845

e23= 0.32922 e24= 0.06584 e34= 0.32922

Significant regression coefficients:

b0 =75.73062 b3 =-2.51687

e0 = 3.68083 e3 = 1.64612

------------------------------------------------------------------

N X1 X2 X3 X4 Yavg Yr styp

------------------------------------------------------------------

1 2.500 60.000 3.000 60.000 67.52 68.18 0.98

2 1.500 60.000 3.000 60.000 67.58 68.18 0.89

3 2.500 50.000 3.000 60.000 67.55 68.18 0.93

4 1.500 50.000 3.000 60.000 67.45 68.18 1.08

5 2.500 60.000 1.000 60.000 73.28 73.21 0.09

6 1.500 60.000 1.000 60.000 73.44 73.21 0.31

7 2.500 50.000 1.000 60.000 73.23 73.21 0.02

8 1.500 50.000 1.000 60.000 73.28 73.21 0.09

9 2.500 60.000 3.000 50.000 67.38 68.18 1.19

10 1.500 60.000 3.000 50.000 67.23 68.18 1.41

11 2.500 50.000 3.000 50.000 73.48 68.18 7.21

12 1.500 50.000 3.000 50.000 67.25 68.18 1.38

13 2.500 60.000 1.000 50.000 73.18 73.21 0.05

14 1.500 60.000 1.000 50.000 73.11 73.21 0.14

15 2.500 50.000 1.000 50.000 73.07 73.21 0.20

16 1.500 50.000 1.000 50.000 73.12 73.21 0.13

------------------------------------------------------------------

min 1.500 50.000 3.000 50.000 68.18

max 1.500 50.000 1.000 50.000 73.21

Statistical indicators:

Student’s criterion tcr= 4.304

variance of error of experience and inadequacy s2y= 2.3409 s2ag= 2.3089

standard deviation sy = 1.5300 sag = 1.5195

degrees of freedom Ns2y= 2 Ns2ag= 14

Fisher’s criterion Fr= 1.01 Fcr= 3.74

CALCULATION OF REGRESSION COEFFICIENTS BY THE LEAST SQUARES METHOD FOR A LINEAR PLAN WITH INTERFACTOR INTERACTIONS

Number of experiments N= 16, coefficients KK=11, factors KF= 4

Х1 - Cо*10^-6, mg/unit.; Х2 - Tw, °C С; Х3 - Ccp, %; Х4 - Td, °C

У15- Fiber, %

Number of replicates of experiments m = 1 m0= 0

Regression coefficients (b) and their confidence errors (e):

b0 = 2.09125 b1 =-0.66875 b2 = 0.02950 b3 = 0.50563

b4 = 0.03550 b12= 0.01125 b13=-0.01375 b14= 0.00125

b23= 0.00387 b24=-0.00103 b34= 0.00837

e0 =19.83629 e1 = 4.43015 e2 = 0.33590 e3 = 2.26824

e4 = 0.33590 e12= 0.05638 e13= 0.28188 e14= 0.05638

e23= 0.02819 e24= 0.00564 e34= 0.02819

Significant regression coefficients:

b0 = 2.60313 b3 = 1.15188

e0 = 0.31516 e3 = 0.14094

------------------------------------------------------------------

N X1 X2 X3 X4 Yavg Yr styp

------------------------------------------------------------------

1 2.500 60.000 3.000 60.000 6.07 6.06 0.19

2 1.500 60.000 3.000 60.000 6.09 6.06 0.51

3 2.500 50.000 3.000 60.000 6.13 6.06 1.16

4 1.500 50.000 3.000 60.000 6.08 6.06 0.35

5 2.500 60.000 1.000 60.000 3.71 3.76 1.21

6 1.500 60.000 1.000 60.000 3.69 3.76 1.76

7 2.500 50.000 1.000 60.000 3.68 3.76 2.04

8 1.500 50.000 1.000 60.000 3.74 3.76 0.40

9 2.500 60.000 3.000 50.000 6.18 6.06 1.96

10 1.500 60.000 3.000 50.000 6.04 6.06 0.31

11 2.500 50.000 3.000 50.000 5.81 6.06 4.28

12 1.500 50.000 3.000 50.000 6.07 6.06 0.19

13 2.500 60.000 1.000 50.000 3.83 3.76 1.96

14 1.500 60.000 1.000 50.000 3.78 3.76 0.66

15 2.500 50.000 1.000 50.000 3.81 3.76 1.44

16 1.500 50.000 1.000 50.000 3.80 3.76 1.18

------------------------------------------------------------------

min 1.500 50.000 1.000 50.000 3.76

max 1.500 50.000 3.000 50.000 6.06

Statistical indicators:

Student’s criterion tcr= 4.304

variance of error of experience and inadequacy s2y= 0.0172 s2ag= 0.0076

standard deviation sy = 0.1310 sag = 0.0875

degrees of freedom Ns2y= 2 Ns2ag= 14

Fisher’s criterion Fr= 2.24 Fcr= 3.74

CALCULATION OF REGRESSION COEFFICIENTS BY THE LEAST SQUARES METHOD FOR A LINEAR PLAN WITH INTERFACTOR INTERACTIONS

Number of experiments N= 16, coefficients KK=11, factors KF= 4

Х1 - Cо*10^-6, mg/unit.; Х2 - Tw, °C С; Х3 - Ccp, %; Х4 - Td, °C

У16 - Ash, %

Number of replicates of experiments m = 1 m0= 0

Regression coefficients (b) and their confidence errors (e):

b0 =-1.40375 b1 =-0.24375 b2 = 0.04750 b3 = 0.47562

b4 = 0.04000 b12=-0.00175 b13=-0.00875 b14= 0.00625

b23=-0.00063 b24=-0.00082 b34=-0.00412

e0 = 6.51115 e1 = 1.45417 e2 = 0.11026 e3 = 0.74454

e4 = 0.11026 e12= 0.01851 e13= 0.09253 e14= 0.01851

e23= 0.00925 e24= 0.00185 e34= 0.00925

Significant regression coefficients:

b0 = 0.92063 b3 = 0.19687

e0 = 0.10345 e3 = 0.04626

------------------------------------------------------------------

N X1 X2 X3 X4 Yavg Yr styp

------------------------------------------------------------------

1 2.500 60.000 3.000 60.000 1.46 1.51 3.51

2 1.500 60.000 3.000 60.000 1.41 1.51 7.18

3 2.500 50.000 3.000 60.000 1.54 1.51 1.87

4 1.500 50.000 3.000 60.000 1.53 1.51 1.23

5 2.500 60.000 1.000 60.000 1.09 1.12 2.52

6 1.500 60.000 1.000 60.000 1.14 1.12 1.97

7 2.500 50.000 1.000 60.000 1.18 1.12 5.30

8 1.500 50.000 1.000 60.000 1.12 1.12 0.22

9 2.500 60.000 3.000 50.000 1.54 1.51 1.87

10 1.500 60.000 3.000 50.000 1.57 1.51 3.74

11 2.500 50.000 3.000 50.000 1.46 1.51 3.51

12 1.500 50.000 3.000 50.000 1.58 1.51 4.35

13 2.500 60.000 1.000 50.000 1.07 1.12 4.44

14 1.500 60.000 1.000 50.000 1.13 1.12 1.11

15 2.500 50.000 1.000 50.000 1.12 1.12 0.22

16 1.500 50.000 1.000 50.000 1.09 1.12 2.52

------------------------------------------------------------------

min 1.500 50.000 1.000 50.000 1.12

max 1.500 50.000 3.000 50.000 1.51

Statistical indicators:

Student’s criterion tcr= 4.304

variance of error of experience and inadequacy s2y= 0.0018 s2ag= 0.0024

standard deviation sy = 0.0430 sag = 0.0493

degrees of freedom Ns2y= 2 Ns2ag= 14

Fisher’s criterion Fr= 1.31 Fcr= 19.42

CALCULATION OF REGRESSION COEFFICIENTS BY THE LEAST SQUARES METHOD FOR A LINEAR PLAN WITH INTERFACTOR INTERACTIONS

Number of experiments N= 16, coefficients KK=11, factors KF= 4

Х1 - Cо*10^-6, mg/unit.; Х2 - Tw, °C С; Х3 - Ccp, %; Х4 - Td, °C

У17 - Vitamin A, mg/100g

Number of replicates of experiments m = 1 m0= 0

Regression coefficients (b) and their confidence errors (e):

b0 = 0.87875 b1 =-0.07875 b2 =-0.01025 b3 = 0.21687

b4 =-0.01600 b12= 0.00075 b13= 0.01125 b14= 0.00025

b23= 0.00137 b24= 0.00017 b34= 0.00313

e0 = 4.69408 e1 = 1.04836 e2 = 0.07949 e3 = 0.53676

e4 = 0.07949 e12= 0.01334 e13= 0.06671 e14= 0.01334

e23= 0.00667 e24= 0.00133 e34= 0.00667

Significant regression coefficients:

b0 =-0.08312 b3 = 0.48687

e0 = 0.07458 e3 = 0.03335

------------------------------------------------------------------

N X1 X2 X3 X4 Yavg Yr styp

------------------------------------------------------------------

1 2.500 60.000 3.000 60.000 1.44 1.38 4.34

2 1.500 60.000 3.000 60.000 1.43 1.38 3.67

3 2.500 50.000 3.000 60.000 1.37 1.38 0.55

4 1.500 50.000 3.000 60.000 1.34 1.38 2.80

5 2.500 60.000 1.000 60.000 0.37 0.40 9.12

6 1.500 60.000 1.000 60.000 0.42 0.40 3.87

7 2.500 50.000 1.000 60.000 0.39 0.40 3.53

8 1.500 50.000 1.000 60.000 0.38 0.40 6.25

9 2.500 60.000 3.000 50.000 1.39 1.38 0.90

10 1.500 60.000 3.000 50.000 1.35 1.38 2.04

11 2.500 50.000 3.000 50.000 1.33 1.38 3.57

12 1.500 50.000 3.000 50.000 1.37 1.38 0.55

13 2.500 60.000 1.000 50.000 0.44 0.40 8.24

14 1.500 60.000 1.000 50.000 0.43 0.40 6.10

15 2.500 50.000 1.000 50.000 0.39 0.40 3.53

16 1.500 50.000 1.000 50.000 0.41 0.40 1.52

------------------------------------------------------------------

min 1.500 50.000 1.000 50.000 0.40

max 1.500 50.000 3.000 50.000 1.38

Statistical indicators:

Student’s criterion tcr= 4.304

variance of error of experience and inadequacy s2y= 0.0010 s2ag= 0.0011

standard deviation sy = 0.0310 sag = 0.0335

degrees of freedom Ns2y= 2 Ns2ag= 14

Fisher’s criterion Fr= 1.17 Fcr= 19.42

CALCULATION OF REGRESSION COEFFICIENTS BY THE LEAST SQUARES METHOD FOR A LINEAR PLAN WITH INTERFACTOR INTERACTIONS

Number of experiments N= 16, coefficients KK=11, factors KF= 4

Х1 - Cо*10^-6, mg/unit.; Х2 - Tw, °C С; Х3 - Ccp, %; Х4 - Td, °C

У18- Vitamin E, mg/100g

Number of replicates of experiments m = 1 m0= 0

Regression coefficients (b) and their confidence errors (e):

b0 = 1.65625 b1 =-0.44875 b2 = 0.00225 b3 = 0.40062

b4 = 0.00725 b12= 0.00375 b13=-0.02625 b14= 0.00475

b23= 0.00512 b24=-0.00028 b34= 0.00262

e0 =19.98771 e1 = 4.46397 e2 = 0.33847 e3 = 2.28556

e4 = 0.33847 e12= 0.05681 e13= 0.28404 e14= 0.05681

e23= 0.02840 e24= 0.00568 e34= 0.02840

Significant regression coefficients:

b0 = 1.38437 b3 = 0.77438

e0 = 0.31756 e3 = 0.14202

------------------------------------------------------------------

N X1 X2 X3 X4 Yavg Yr styp

------------------------------------------------------------------

1 2.500 60.000 3.000 60.000 3.85 3.71 3.70

2 1.500 60.000 3.000 60.000 3.78 3.71 1.92

3 2.500 50.000 3.000 60.000 3.65 3.71 1.58

4 1.500 50.000 3.000 60.000 3.74 3.71 0.87

5 2.500 60.000 1.000 60.000 2.13 2.16 1.35

6 1.500 60.000 1.000 60.000 2.18 2.16 0.97

7 2.500 50.000 1.000 60.000 2.22 2.16 2.76

8 1.500 50.000 1.000 60.000 2.19 2.16 1.43

9 2.500 60.000 3.000 50.000 3.68 3.71 0.75

10 1.500 60.000 3.000 50.000 3.72 3.71 0.34

11 2.500 50.000 3.000 50.000 3.53 3.71 5.03

12 1.500 50.000 3.000 50.000 3.71 3.71 0.07

13 2.500 60.000 1.000 50.000 2.14 2.16 0.88

14 1.500 60.000 1.000 50.000 2.18 2.16 0.97

15 2.500 50.000 1.000 50.000 2.13 2.16 1.35

16 1.500 50.000 1.000 50.000 2.10 2.16 2.80

------------------------------------------------------------------

min 1.500 50.000 1.000 50.000 2.16

max 1.500 50.000 3.000 50.000 3.71

Statistical indicators:

Student’s criterion tcr= 4.304

variance of error of experience and inadequacy s2y= 0.0174 s2ag= 0.0052

standard deviation sy = 0.1320 sag = 0.0724

degrees of freedom Ns2y= 2 Ns2ag= 14

Fisher’s criterion Fr= 3.32 Fcr= 3.74

CALCULATION OF REGRESSION COEFFICIENTS BY THE LEAST SQUARES METHOD FOR A LINEAR PLAN WITH INTERFACTOR INTERACTIONS

Number of experiments N= 16, coefficients KK=11, factors KF= 4

Х1 - Cо*10^-6, mg/unit.; Х2 - Tw, °C С; Х3 - Ccp, %; Х4 - Td, °C

У19 - Vitamin C, mg/100g

Number of replicates of experiments m = 1 m0= 0

Regression coefficients (b) and their confidence errors (e):

b0 =-2.70250 b1 =-0.16500 b2 = 0.05250 b3 = 0.71875

b4 = 0.05650 b12=-0.00000 b13=-0.02750 b14= 0.00400

b23= 0.00375 b24=-0.00110 b34= 0.00025

e0 = 5.14835 e1 = 1.14981 e2 = 0.08718 e3 = 0.58870

e4 = 0.08718 e12= 0.01463 e13= 0.07316 e14= 0.01463

e23= 0.00732 e24= 0.00146 e34= 0.00732

Significant regression coefficients:

b0 = 0.00000 b3 = 0.91375

e0 = 0.00000 e3 = 0.01636

------------------------------------------------------------------

N X1 X2 X3 X4 Yavg Yr styp

------------------------------------------------------------------

1 2.500 60.000 3.000 60.000 2.76 2.74 0.68

2 1.500 60.000 3.000 60.000 2.74 2.74 0.05

3 2.500 50.000 3.000 60.000 2.72 2.74 0.78

4 1.500 50.000 3.000 60.000 2.78 2.74 1.39

5 2.500 60.000 1.000 60.000 0.92 0.91 0.68

6 1.500 60.000 1.000 60.000 0.92 0.91 0.68

7 2.500 50.000 1.000 60.000 1.10 0.91 16.93

8 1.500 50.000 1.000 60.000 0.98 0.91 6.76

9 2.500 60.000 3.000 50.000 2.74 2.74 0.05

10 1.500 60.000 3.000 50.000 2.73 2.74 0.41

11 2.500 50.000 3.000 50.000 2.63 2.74 4.23

12 1.500 50.000 3.000 50.000 2.71 2.74 1.15

13 2.500 60.000 1.000 50.000 0.94 0.91 2.79

14 1.500 60.000 1.000 50.000 0.97 0.91 5.80

15 2.500 50.000 1.000 50.000 0.93 0.91 1.75

16 1.500 50.000 1.000 50.000 0.91 0.91 0.41

------------------------------------------------------------------

min 1.500 50.000 1.000 50.000 0.91

max 1.500 50.000 3.000 50.000 2.74

Statistical indicators:

Student’s criterion tcr= 4.304

variance of error of experience and inadequacy s2y= 0.0012 s2ag= 0.0039

standard deviation sy = 0.0340 sag = 0.0628

degrees of freedom Ns2y= 2 Ns2ag= 15

Fisher’s criterion Fr= 3.41 Fcr= 19.43

CALCULATION OF REGRESSION COEFFICIENTS BY THE LEAST SQUARES METHOD FOR A LINEAR PLAN WITH INTERFACTOR INTERACTIONS

Number of experiments N= 16, coefficients KK=11, factors KF= 4

Х1 - Cо*10^-6, mg/unit.; Х2 - Tw, °C С; Х3 - Ccp, %; Х4 - Td, °C

У20 - Beta-carotene, mg/100g

Number of replicates of experiments m = 1 m0= 0

Regression coefficients (b) and their confidence errors (e):

b0 =-0.33000 b1 =-1.58250 b2 = 0.04675 b3 = 3.13000

b4 = 0.03375 b12= 0.01600 b13=-0.06250 b14= 0.01400

b23= 0.00325 b24=-0.00140 b34= 0.00875

e0 =19.83629 e1 = 4.43015 e2 = 0.33590 e3 = 2.26824

e4 = 0.33590 e12= 0.05638 e13= 0.28188 e14= 0.05638

e23= 0.02819 e24= 0.00564 e34= 0.02819

Significant regression coefficients:

b0 = 0.00000 b3 = 3.66400

e0 = 0.00000 e3 = 0.06303

------------------------------------------------------------------

N X1 X2 X3 X4 Yavg Yr styp

------------------------------------------------------------------

1 2.500 60.000 3.000 60.000 11.04 10.99 0.43

2 1.500 60.000 3.000 60.000 11.02 10.99 0.25

3 2.500 50.000 3.000 60.000 11.07 10.99 0.70

4 1.500 50.000 3.000 60.000 11.06 10.99 0.61

5 2.500 60.000 1.000 60.000 3.68 3.66 0.43

6 1.500 60.000 1.000 60.000 3.64 3.66 0.66

7 2.500 50.000 1.000 60.000 3.59 3.66 2.06

8 1.500 50.000 1.000 60.000 3.61 3.66 1.50

9 2.500 60.000 3.000 50.000 11.08 10.99 0.79

10 1.500 60.000 3.000 50.000 11.06 10.99 0.61

11 2.500 50.000 3.000 50.000 10.54 10.99 4.29

12 1.500 50.000 3.000 50.000 11.07 10.99 0.70

13 2.500 60.000 1.000 50.000 3.72 3.66 1.51

14 1.500 60.000 1.000 50.000 3.71 3.66 1.24

15 2.500 50.000 1.000 50.000 3.67 3.66 0.16

16 1.500 50.000 1.000 50.000 3.68 3.66 0.43

------------------------------------------------------------------

min 1.500 50.000 1.000 50.000 3.66

max 1.500 50.000 3.000 50.000 10.99

Statistical indicators:

Student’s criterion tcr= 4.304

variance of error of experience and inadequacy s2y= 0.0172 s2ag= 0.0168

standard deviation sy = 0.1310 sag = 0.1294

degrees of freedom Ns2y= 2 Ns2ag= 15

Fisher’s criterion Fr= 1.02 Fcr= 3.68

CALCULATION OF REGRESSION COEFFICIENTS BY THE LEAST SQUARES METHOD FOR A LINEAR PLAN WITH INTERFACTOR INTERACTIONS

Number of experiments N= 16, coefficients KK=11, factors KF= 4

Х1 - Cо*10^-6, mg/unit.; Х2 - Tw, °C С; Х3 - Ccp, %; Х4 - Td, °C

У22 - Mineral content – Potassium (K)

Number of replicates of experiments m = 1 m0= 0

Regression coefficients (b) and their confidence errors (e):

b0 =573.75125 b1 =-127.08875 b2 =-4.24925 b3 = 9.10437

b4 =-4.58900 b12= 1.35075 b13=-5.59375 b14= 1.11025

b23= 0.21287 b24= 0.02778 b34= 0.97813

e0 =2321.29967 e1 =518.42910 e2 =39.30837 e3 =265.43658

e4 =39.30837 e12= 6.59737 e13=32.98685 e14= 6.59737

e23= 3.29869 e24= 0.65974 e34= 3.29869

Significant regression coefficients:

b0 =188.19938 b3 =63.42187

e0 =36.88042 e3 =16.49343

------------------------------------------------------------------

N X1 X2 X3 X4 Yavg Yr styp

------------------------------------------------------------------

1 2.500 60.000 3.000 60.000 398.52 378.47 5.03

2 1.500 60.000 3.000 60.000 386.53 378.47 2.09

3 2.500 50.000 3.000 60.000 379.33 378.47 0.23

4 1.500 50.000 3.000 60.000 391.35 378.47 3.29

5 2.500 60.000 1.000 60.000 258.16 251.62 2.53

6 1.500 60.000 1.000 60.000 250.16 251.62 0.58

7 2.500 50.000 1.000 60.000 251.73 251.62 0.04

8 1.500 50.000 1.000 60.000 249.18 251.62 0.98

9 2.500 60.000 3.000 50.000 368.17 378.47 2.80

10 1.500 60.000 3.000 50.000 373.01 378.47 1.46

11 2.500 50.000 3.000 50.000 350.81 378.47 7.88

12 1.500 50.000 3.000 50.000 380.00 378.47 0.40

13 2.500 60.000 1.000 50.000 251.10 251.62 0.21

14 1.500 60.000 1.000 50.000 250.92 251.62 0.28

15 2.500 50.000 1.000 50.000 250.84 251.62 0.31

16 1.500 50.000 1.000 50.000 250.88 251.62 0.30

------------------------------------------------------------------

min 1.500 50.000 1.000 50.000 251.62

max 1.500 50.000 3.000 50.000 378.47

Statistical indicators:

Student’s criterion tcr= 4.304

variance of error of experience and inadequacy s2y=235.0089 s2ag=113.5502

standard deviation sy = 15.3300 sag = 10.6560

degrees of freedom Ns2y= 2 Ns2ag= 14

Fisher’s criterion Fr= 2.07 Fcr= 3.74

CALCULATION OF REGRESSION COEFFICIENTS BY THE LEAST SQUARES METHOD FOR A LINEAR PLAN WITH INTERFACTOR INTERACTIONS

Number of experiments N= 16, coefficients KK=11, factors KF= 4

Х1 - Cо*10^-6, mg/unit.; Х2 - Tw, °C С; Х3 - Ccp, %; Х4 - Td, °C

У23 - Mineral content – Magnesium (Mg)

Number of replicates of experiments m = 1 m0= 0

Regression coefficients (b) and their confidence errors (e):

b0 =64.65250 b1 = 2.21500 b2 =-0.33250 b3 =-15.31375

b4 =-0.83500 b12=-0.07500 b13= 0.43500 b14= 0.03000

b23= 0.05025 b24= 0.00875 b34= 0.23175

e0 =124.16606 e1 =27.73072 e2 = 2.10260 e3 =14.19817

e4 = 2.10260 e12= 0.35289 e13= 1.76446 e14= 0.35289

e23= 0.17645 e24= 0.03529 e34= 0.17645

Significant regression coefficients:

b0 =26.38875 b3 =-5.21750 b34= 0.11425

e0 = 1.97273 e3 = 4.42877 e34= 0.07891

------------------------------------------------------------------

N X1 X2 X3 X4 Yavg Yr styp

------------------------------------------------------------------

1 2.500 60.000 3.000 60.000 32.95 31.30 5.00

2 1.500 60.000 3.000 60.000 32.13 31.30 2.58

3 2.500 50.000 3.000 60.000 31.47 31.30 0.54

4 1.500 50.000 3.000 60.000 29.83 31.30 4.93

5 2.500 60.000 1.000 60.000 27.65 28.03 1.36

6 1.500 60.000 1.000 60.000 27.61 28.03 1.51

7 2.500 50.000 1.000 60.000 26.93 28.03 4.07

8 1.500 50.000 1.000 60.000 26.39 28.03 6.20

9 2.500 60.000 3.000 50.000 28.09 27.87 0.77

10 1.500 60.000 3.000 50.000 28.17 27.87 1.05

11 2.500 50.000 3.000 50.000 27.93 27.87 0.20

12 1.500 50.000 3.000 50.000 26.13 27.87 6.67

13 2.500 60.000 1.000 50.000 27.85 26.88 3.47

14 1.500 60.000 1.000 50.000 27.69 26.88 2.91

15 2.500 50.000 1.000 50.000 27.74 26.88 3.09

16 1.500 50.000 1.000 50.000 27.78 26.88 3.23

------------------------------------------------------------------

min 1.500 50.000 1.000 50.000 26.88

max 1.500 50.000 3.000 60.000 31.30

Statistical indicators:

Student’s criterion tcr= 4.304

variance of error of experience and inadequacy s2y= 0.6724 s2ag= 1.2377

standard deviation sy = 0.8200 sag = 1.1125

degrees of freedom Ns2y= 2 Ns2ag= 13

Fisher’s criterion Fr= 1.84 Fcr= 19.42

CALCULATION OF REGRESSION COEFFICIENTS BY THE LEAST SQUARES METHOD FOR A LINEAR PLAN WITH INTERFACTOR INTERACTIONS

Number of experiments N= 16, coefficients KK=11, factors KF= 4

Х1 - Cо*10^-6, mg/unit.; Х2 - Tw, °C С; Х3 - Ccp, %; Х4 - Td, °C

У24 - Mineral content – Iron (Fe)

Number of replicates of experiments m = 1 m0= 0

Regression coefficients (b) and their confidence errors (e):

b0 =-1.52612 b1 =-0.05712 b2 = 0.03730 b3 = 0.14969

b4 = 0.05052 b12= 0.00057 b13= 0.04212 b14=-0.00058

b23= 0.00021 b24=-0.00066 b34=-0.00221

e0 =10.75096 e1 = 2.40107 e2 = 0.18205 e3 = 1.22935

e4 = 0.18205 e12= 0.03056 e13= 0.15278 e14= 0.03056

e23= 0.01528 e24= 0.00306 e34= 0.01528

Significant regression coefficients:

b0 = 1.24172 b13= 0.05180

e0 = 0.15368 e13= 0.03334

------------------------------------------------------------------

N X1 X2 X3 X4 Yavg Yr styp

------------------------------------------------------------------

1 2.500 60.000 3.000 60.000 1.67 1.63 2.38

2 1.500 60.000 3.000 60.000 1.53 1.47 3.61

3 2.500 50.000 3.000 60.000 1.62 1.63 0.63

4 1.500 50.000 3.000 60.000 1.60 1.47 8.00

5 2.500 60.000 1.000 60.000 1.36 1.37 0.83

6 1.500 60.000 1.000 60.000 1.40 1.32 5.76

7 2.500 50.000 1.000 60.000 1.37 1.37 0.09

8 1.500 50.000 1.000 60.000 1.39 1.32 5.08

9 2.500 60.000 3.000 50.000 1.60 1.63 1.89

10 1.500 60.000 3.000 50.000 1.55 1.47 4.85

11 2.500 50.000 3.000 50.000 1.54 1.63 5.86

12 1.500 50.000 3.000 50.000 1.47 1.47 0.33

13 2.500 60.000 1.000 50.000 1.28 1.37 7.13

14 1.500 60.000 1.000 50.000 1.31 1.32 0.72

15 2.500 50.000 1.000 50.000 1.26 1.37 8.83

16 1.500 50.000 1.000 50.000 1.23 1.32 7.27

------------------------------------------------------------------

min 1.500 50.000 1.000 50.000 1.32

max 2.500 50.000 3.000 50.000 1.63

Statistical indicators:

Student’s criterion tcr= 4.304

variance of error of experience and inadequacy s2y= 0.0050 s2ag= 0.0054

standard deviation sy = 0.0710 sag = 0.0738

degrees of freedom Ns2y= 2 Ns2ag= 14

Fisher’s criterion Fr= 1.08 Fcr= 19.42

CALCULATION OF REGRESSION COEFFICIENTS BY THE LEAST SQUARES METHOD FOR A LINEAR PLAN WITH INTERFACTOR INTERACTIONS

Number of experiments N= 16, coefficients KK=11, factors KF= 4

Х1 - Cо*10^-6, mg/unit.; Х2 - Tw, °C С; Х3 - Ccp, %; Х4 - Td, °C

У25 - Mineral content – Zinc (Zn)

Number of replicates of experiments m = 1 m0= 0

Regression coefficients (b) and their confidence errors (e):

b0 = 1.81750 b1 =-0.20000 b2 =-0.00925 b3 =-0.41625

b4 =-0.01475 b12= 0.00150 b13= 0.01750 b14= 0.00150

b23= 0.00050 b24= 0.00010 b34= 0.00700

e0 = 5.45119 e1 = 1.21745 e2 = 0.09231 e3 = 0.62333

e4 = 0.09231 e12= 0.01549 e13= 0.07746 e14= 0.01549

e23= 0.00775 e24= 0.00155 e34= 0.00775

Significant regression coefficients:

b0 = 0.73000 b3 =-0.21625 b34= 0.00450

e0 = 0.08661 e3 = 0.19443 e34= 0.00346

------------------------------------------------------------------

N X1 X2 X3 X4 Yavg Yr styp

------------------------------------------------------------------

1 2.500 60.000 3.000 60.000 0.92 0.89 3.13

2 1.500 60.000 3.000 60.000 0.87 0.89 2.44

3 2.500 50.000 3.000 60.000 0.91 0.89 2.06

4 1.500 50.000 3.000 60.000 0.89 0.89 0.14

5 2.500 60.000 1.000 60.000 0.78 0.78 0.48

6 1.500 60.000 1.000 60.000 0.77 0.78 1.79

7 2.500 50.000 1.000 60.000 0.73 0.78 7.36

8 1.500 50.000 1.000 60.000 0.78 0.78 0.48

9 2.500 60.000 3.000 50.000 0.75 0.76 0.83

10 1.500 60.000 3.000 50.000 0.77 0.76 1.79

11 2.500 50.000 3.000 50.000 0.75 0.76 0.83

12 1.500 50.000 3.000 50.000 0.73 0.76 3.60

13 2.500 60.000 1.000 50.000 0.74 0.74 0.17

14 1.500 60.000 1.000 50.000 0.75 0.74 1.50

15 2.500 50.000 1.000 50.000 0.76 0.74 2.80

16 1.500 50.000 1.000 50.000 0.78 0.74 5.29

------------------------------------------------------------------

min 1.500 50.000 1.000 50.000 0.74

max 1.500 50.000 3.000 60.000 0.89

Statistical indicators:

Student’s criterion tcr= 4.304

variance of error of experience and inadequacy s2y= 0.0013 s2ag= 0.0006

standard deviation sy = 0.0360 sag = 0.0248

degrees of freedom Ns2y= 2 Ns2ag= 13

Fisher’s criterion Fr= 2.11 Fcr= 3.81
